# The plasma membrane calcium ATPase 4 signalling in cardiac fibroblasts mediates cardiomyocyte hypertrophy

**DOI:** 10.1038/ncomms11074

**Published:** 2016-03-29

**Authors:** Tamer M. A. Mohamed, Riham Abou-Leisa, Nicholas Stafford, Arfa Maqsood, Min Zi, Sukhpal Prehar, Florence Baudoin-Stanley, Xin Wang, Ludwig Neyses, Elizabeth J. Cartwright, Delvac Oceandy

**Affiliations:** 1 Institute of Cardiovascular Sciences, University of Manchester, AV Hill Building, Manchester M13 9PT, UK; 2 J David Gladstone Research Institutes, San Francisco, California 94158, USA; 3 Faculty of Pharmacy, Zagazig University, Zagazig 44519, Egypt; 4 Faculty of Life Sciences, University of Manchester, Manchester M13 9PT, UK

## Abstract

The heart responds to pathological overload through myocyte hypertrophy. Here we show that this response is regulated by cardiac fibroblasts via a paracrine mechanism involving plasma membrane calcium ATPase 4 (PMCA4). *Pmca4* deletion in mice, both systemically and specifically in fibroblasts, reduces the hypertrophic response to pressure overload; however, knocking out *Pmca4* specifically in cardiomyocytes does not produce this effect. Mechanistically, cardiac fibroblasts lacking PMCA4 produce higher levels of secreted frizzled related protein 2 (sFRP2), which inhibits the hypertrophic response in neighbouring cardiomyocytes. Furthermore, we show that treatment with the PMCA4 inhibitor aurintricarboxylic acid (ATA) inhibits and reverses cardiac hypertrophy induced by pressure overload in mice. Our results reveal that PMCA4 regulates the development of cardiac hypertrophy and provide proof of principle for a therapeutic approach to treat this condition.

## Abstract (web summary)

Restricting hypertrophic heart growth in response to pathologic overload is an unmet therapeutic need. Here, the authors show that blocking Ca^2+^ signaling controlled by the transport protein PMCA4 in cardiac fibroblasts enhances secretion of a potent Wnt signaling inhibitor, sFRP2, and prevents the development of pathologic cardiac hypertrophy in mice.

## Graphical abstract

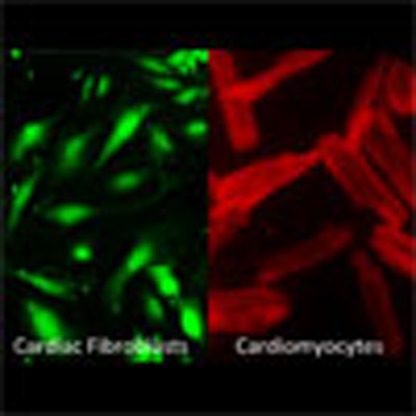

Cardiac overload due to high blood pressure, myocardial infarction, aortic stenosis and myofilament or cytoskeletal mutations leads to pathological hypertrophy and eventually heart failure^1^,^2^,^3^. Controlling hypertrophic growth is important, as it significantly reduces the risk of developing heart failure and sudden death^4^. Several treatment modalities are commonly used to control the extra-cardiac factors that may contribute to hypertrophic growth, notably blood pressure^5^; however, no treatment has directly targeted the intra-cardiac factors. Therefore, the investigation of the intra-cardiac mechanisms governing hypertrophic growth is pivotal for developing novel pathophysiologial and therapeutic concepts.

Cardiac fibroblasts have recently emerged as one of the main factors in the regulation of various pathological processes in the heart. Cardiac fibroblasts play key roles in maintaining extracellular matrix homeostasis (reviewed in ref. 6). These cells are commonly understood to be heavily involved in the development of myocardial fibrosis through cell proliferation and secretion of extracellular matrix. However, recent knowledge suggests that cardiac fibroblasts are actively involved in the regulation of a number of signalling pathways in the heart, including those implicated in cardiac hypertrophy and remodelling^6^. These cells interact with cardiomyocytes via paracrine mechanisms and/or direct cell–cell interactions^7^. Examples of factors secreted by cardiac fibroblasts that may mediate cardiomyocyte hypertrophy include growth factors (for example, insulin-like growth factor 1 (IGF1))^8^ and microRNAs^9^.

Calcium is an important signal transducer and is essential in the regulation of key cellular processes such as growth, survival and gene expression^10^. Although regulation of the calcium signals in cardiomyocytes is well studied, the calcium signalling mechanism in cardiac fibroblasts is relatively unknown. A recent study has indicated that regulation of intracellular calcium might influence cardiac fibroblasts proliferation rate and hence the development of fibrosis^11^; however, it is not known whether intracellular calcium in fibroblasts mediates cardiac hypertrophy.

Here we show that the plasma membrane calcium ATPase isoform 4 (PMCA4) regulates the calcium signal in cardiac fibroblasts, which is important in the regulation of cardiac hypertrophy. Genetic ablation and pharmacological inhibition of PMCA4 enhances the production of secreted frizzled related protein 2 (sFRP2) by cardiac fibroblasts. sFRP2 is a potent inhibitor of the Wnt pathway and has been described as having potent protective effects against myocardial injury^12^. We also show that targeting PMCA4 by a novel inhibitor is beneficial to the progression of cardiac hypertrophy probably through potentiation of sFRP2 production.

## Results

### sFRP2 expression is elevated in *PMCA4* ^*−/−*^ cardiac fibroblasts

**Figure 1:**
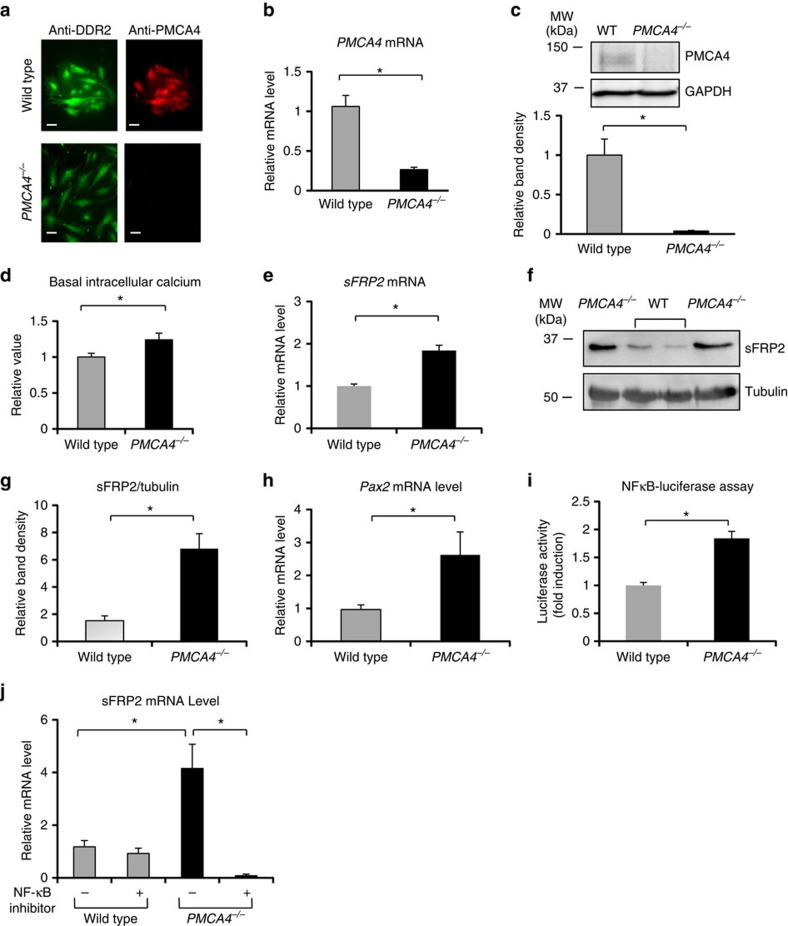
*Pmca4* gene ablation increased sFRP2 expression in ACFs. (**a**) Immunofluorescence analysis of ACFs isolated from WT and *PMCA4*^*−/−*^ mice. Cells were stained with anti-DDR2 (fibroblasts marker) and anti-PMCA4 antibodies (scale bars, 25 μm). (**b**) qRT–PCR analysis showing a significant reduction in *Pmca4* level in ACFs of *PMCA4*^*−/−*^ mice (*n*=4; *P<0.05). (**c**) Western blotting to detect PMCA4 protein level in ACFs and quantification of band density showing significant reduction of PMCA4 expression in *PMCA4*^*−/−*^ cardiac fibroblasts (*n*=4; *P<0.05). (**d**) Analysis of basal intracellular calcium showed significantly elevated intracellular calcium level in *PMCA4*^*−/−*^ cardiac fibroblasts (*n*=4; *P<0.05). (**e**) Real-time RT–PCR and (**f**) western blot analysis, to examine sFRP2 expression in cardiac fibroblasts. (**g**) Quantification of band density supported the qRT–PCR analysis, showing significantly increased sFRP2 expression in *PMCA4*^*−/−*^ fibroblasts (*n*=5; *P<0.05. (**h**) Expression of Pax2 was also elevated in *PMCA4*^*−/−*^ fibroblasts (*n*=4; *P<0.05). (**i**) Using adenovirus-mediated NF-κB luciferase reporter construct, we detected a higher level of NF-κB activity in fibroblasts lacking PMCA4 (*n*=4; *P<0.05). (**j**) Treatment with NF-κB inhibitor (BAY11–7082) at 10 μM for 24 h remarkably reduced sFRP2 mRNA level in *PMCA4*^*−/−*^ fibroblasts as detected by qRT–PCR (*n*=4; *P<0.05). Student's *t*-test was used in all statistical tests above. All error bars represent the s.e.m.

We first studied whether genetic ablation of *Pmca4* in cardiac fibroblasts modified intracellular calcium. PMCA4 was expressed in mouse adult cardiac fibroblasts (ACFs) and its expression was significantly reduced in cardiac fibroblasts isolated from *PMCA4*^*−/−*^ mice as detected by immunofluorescence, quantitative reverse transcriptase–PCR (qRT–PCR) and western blot analyses (Fig. 1a–c). We examined basal intracellular calcium in these cells using the calcium sensitive dye fluo-3 and found that it was 25% higher in *PMCA4*^*−/−*^ fibroblasts compared with wild type (WT; Fig. 1d). This finding suggests that PMCA4 plays a key role in maintaining physiological calcium levels in cardiac fibroblasts.

As calcium mediates multiple signalling pathways and gene expression, we investigated the transcription profile in *PMCA4*^*−/−*^ fibroblasts. Using an Affymetrix microarray GeneChip technology, we first examined the messenger RNA expression profile of *PMCA4*^*−/−*^ fibroblasts. Interestingly, we found that several genes involved in regulating Wnt signalling were elevated in *PMCA4*^*−/−*^ fibroblasts, such as sFRP2 and IGF-binding protein (IGFBP) 4 and 5 (Supplementary Fig. 1A). qRT–PCR and western blots analyses confirmed that sFRP2 mRNA and protein were significantly and consistently elevated in *PMCA4*^*−/−*^ cardiac fibroblasts (Fig. 1e–g). In addition, qRT–PCR analysis showed that *IGFBP4* and *IGFBP5* were also elevated in *PMCA4*^*−/−*^ fibroblasts (Supplementary Fig. 1B,C). However, in this study we focused on sFRP2, as it is known that this molecule plays an essential role in mediating cardiac remodelling^12^,^13^.

sFRP2 expression is induced by the transcription factor Pax2 (ref. 14). We therefore analysed *Pax2* expression and found it to be significantly elevated in *PMCA4*^*−/−*^ cardiac fibroblasts (Fig. 1h). To further investigate the mechanism by which PMCA4 regulates sFRP2 expression, we then focused on nuclear factor-κB (NF-κB) signalling because: (i) NF-κB regulates *Pax2* expression^15^ and (ii) PMCA4 has been demonstrated to be an upstream regulator of NF-κB^16^. Using an adenoviral-mediated NF-κB-luciferase reporter construct, we detected significantly higher NF-κB activity in *PMCA4*^*−/−*^ fibroblasts (Fig. 1i). This increase might be due to the elevated intracellular calcium in *PMCA4*^*−/−*^ fibroblasts, because NF-κB activity has been associated with intracellular calcium levels^17^. To further confirm if NF-κB signalling regulates sFRP2 expression, we treated fibroblasts with the NF-κB inhibitor BAY11–7082 (10 μM). As shown in Fig. 1j, the level of sFRP2 expression in *PMCA4*^*−/−*^ fibroblasts was significantly reduced following treatment with BAY11–7082. Together, our data suggested that signalling via NF-κB and Pax2 pathways may be responsible for the raised sFRP2 expression in these cells.

### *PMCA4* *^−/−^* fibroblast conditioned medium attenuates hypertrophy

**Figure 2:**
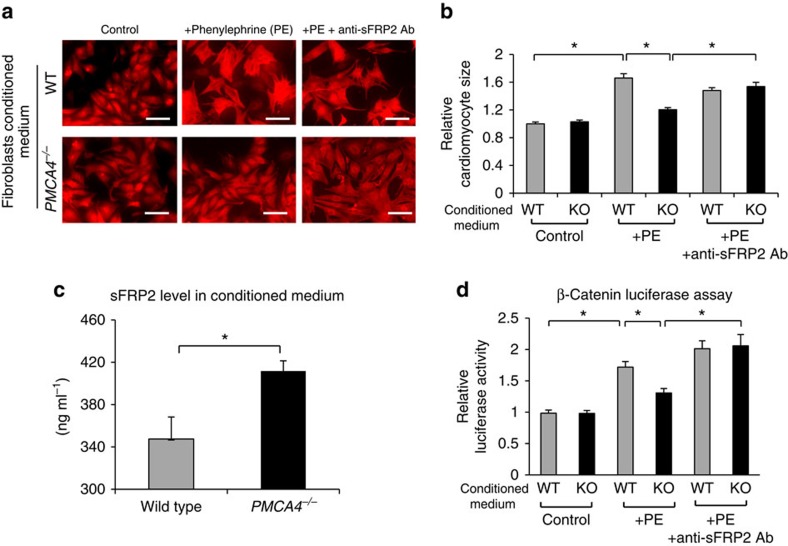
*PMCA4*^*−/−*^ fibroblast conditioned medium exerts an anti-hypertrophic effect. (**a**) Representative images of NRCMs cultured in conditioned medium of either WT or *PMCA4*^*−/−*^ cardiac fibroblasts. NRCMs were treated with phenylephrine (PE), 30 μM or PE with anti-sFRP2 antibody (0.2 μg ml^−1^) for 72 h. Cells were then stained with α-actinin antibody, to specifically visualize cardiomyocytes. Scale bar, 25 μm. (**b**) Measurement of cell surface area indicated that treatment with *PMCA4*^*−/−*^ fibroblasts conditioned medium (KO) significantly reduced PE-induced hypertrophy (results were from three independent experiments conducted in triplicate; a minimum of 100 cells were measured per replicate, *P<0.05, one-way analysis of variance (ANOVA) followed by *post-hoc* multiple comparison). Addition of anti-sFRP2 antibody abolished the anti-hypertrophic effect of *PMCA4*^*−/−*^ conditioned medium. (**c**) ELISA analysis showed a significantly higher sFRP2 level in *PMCA4*^*−/−*^ conditioned medium (*n*=4 in each group; *P<0.05, Student's *t*-test). (**d**) Using an adenovirus expressing the TCF/LEF-luciferase construct, we detected activation of the Wnt/β-catenin pathway. Consistent with this observation, conditioned medium from *PMCA4*^*−/−*^ fibroblasts inhibited PE-induced Wnt/β-catenin activation, which was reversed by treatment with anti-sFRP2 antibody (*n*=3 independent experiments; *P<0.05, one-way ANOVA followed by *post-hoc* multiple comparison). All error bars represent the s.e.m.

sFRP2 is a potent inhibitor of Wnt signalling, which plays key roles in mediating cardiac growth and pathological hypertrophy^18^. Therefore, we hypothesized that higher sFRP2 production by *PMCA4*^*−/−*^ cardiac fibroblasts may protect against cardiomyocyte hypertrophy via a paracrine mechanism. To test this hypothesis, we performed *in vitro* experiments using isolated neonatal rat cardiomyocytes (NRCMs). After 2 days of culture in normal medium, we treated NRCMs with conditioned medium from either WT or *PMCA4*^*−/−*^ ACFs. We then induced cellular hypertrophy by stimulating cells with phenylephrine (30 μM for 72 h) and measured cell size, and found that NRCMs showed a significantly less hypertrophic response when treated with conditioned medium from *PMCA4*^*−/−*^ fibroblasts compared with WT fibroblasts (Fig. 2a,b). Analysis of the levels of atrial natriuretic peptide (ANP) and brain natriuretic peptide (BNP) expression also supported this finding (Supplementary Fig. 2A,B). This data indicated that *PMCA4*^*−/−*^ fibroblasts might secrete a factor that represses hypertrophy. To test whether sFRP2 was the important factor, we conducted rescue experiments by inhibiting sFRP2 activity with an anti-sFRP2 antibody added to the conditioned medium. Treating the cells with anti-sFRP2 abolished the anti-hypertrophic effect of the conditioned medium from *PMCA4*^*−/−*^ fibroblasts (Fig. 2a,b). Furthermore, we performed enzyle-linked immunosorbent assay analysis of the conditioned medium, which showed that the level of sFRP2 was significantly higher in conditioned medium from *PMCA4*^*−/−*^ fibroblasts compared with WT fibroblasts (Fig. 2c). To examine whether Wnt signalling was affected, we generated adenovirus carrying the Wnt-luciferase reporter construct^19^. Using this construct, we showed that in cells treated with conditioned medium from *PMCA4*^*−/−*^ fibroblasts, the phenylephrine-induced Wnt signalling was significantly lower compared with cells in conditioned medium from WT fibroblasts (Fig. 2d). Consistently, treatment with anti-sFRP2 antibody abolished the difference in Wnt signal activation (Fig. 2d). These results demonstrated that *PMCA4*^*−/−*^ fibroblasts protect against phenylephrine-induced cardiomyocyte hypertrophy, possibly by secreting sFRP2. We also tested the anti-hypertrophic effect of *PMCA4*^*−/−*^ fibroblast conditioned medium when used to culture adult rat cardiomyocytes. Consistent results were obtained from experiments using isolated adult rat cardiomyocytes as shown in Supplementary Fig. 2C,D.

### TAC-induced hypertrophy is attenuated in *PMCA4* ^*−/−*^ mice

**Figure 3:**
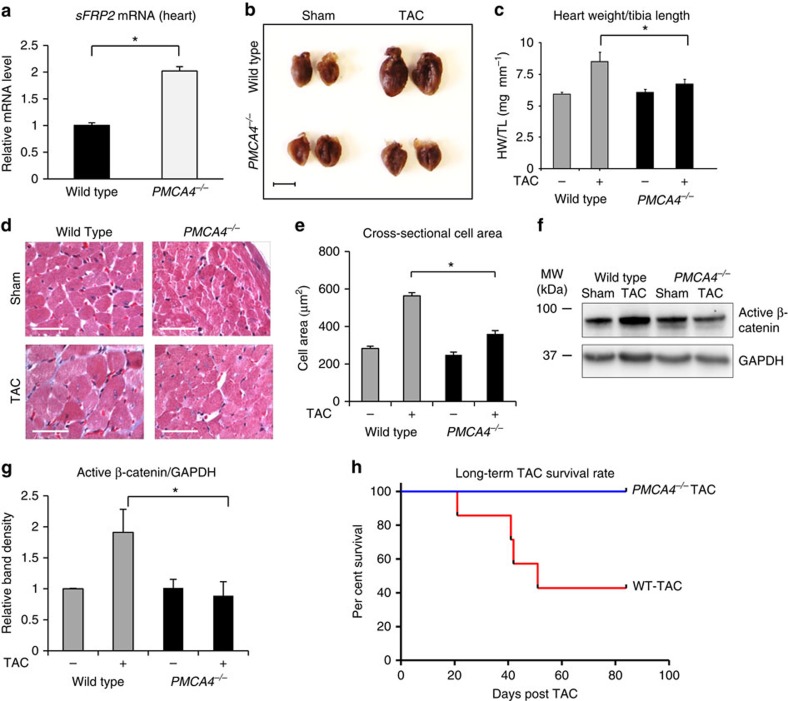
Systemic deletion of *Pmca4* attenuated the hypertrophic response to TAC. (**a**) sFRP2 level was detected in heart tissues using qRT–PCR (WT, *n*=4; *PMCA4*^*−/−*^, *n*=3; *P<0.05, Student's *t*-test). (**b**) Representative image of hearts from WT and *PMCA4*^*−/−*^ mice following TAC (5 weeks) or sham operation (scale bar, 5 mm). (**c**) Measurement of HW/TL ratio showed significant reduction of the hypertrophic response in *PMCA4*^*−/−*^ mice (*n*=10 in each group; *P<0.05, one-way analysis of variance (ANOVA) followed by *post-hoc* multiple comparison). (**d**) Representative heart tissue sections stained with haematoxylin and eosin, and (**e**) measurement of cardiomyocyte cross-sectional area showed smaller cardiomyocyte size in *PMCA4*^*−/−*^ mice after TAC (scale bars, 50 μm,*P<0.05, one-way ANOVA followed by *post-hoc* multiple comparison). (**f**) Western blot analysis of active β-catenin and (**g**) quantification of band density level indicated a higher level of β-catenin activation in *PMCA4*^*−/−*^ mice after TAC (*n*=4 in each group; *P<0.05, one-way ANOVA followed by *post-hoc* multiple comparison). (**h**) When stimulated with long-term (12 weeks) TAC, more *PMCA4*^*−/−*^ mice survived than WT. All of the sham-treated animals survived at 12 weeks following procedure (WT, *n*=7; *PMCA4*^*−/−*^ *n*=5 at the beginning of the experiments). All error bars represent the s.e.m.

To investigate the anti-hypertrophic effect of ablating the *Pmca4* gene *in vivo*, we analysed our PMCA4 global knockout (KO) mice (*PMCA4*^*−/−*^ mice)^20^. In *PMCA4*^*−/−*^ hearts, PMCA4 was completely ablated as we previously described^21^. We found that sFRP2 expression was increased in the hearts of *PMCA4*^*−/−*^ mice (Fig. 3a). We then subjected these mice to transverse aortic constriction (TAC) for 5 weeks, to induce cardiac pressure overload. Systemic deletion of PMCA4 attenuated pathological hypertrophy in response to pressure overload as shown by heart weight/tibia length (HW/TL) ratio, cardiomyocyte cell surface area and echocardiography assessments (Fig. 3b–e and Supplementary Table 1). In these mice, the hypertrophic marker ANP was also lower (Supplementary Fig. 3A). However, we did not observe changes in cardiac contractility in response to TAC, as indicated by the haemodynamic parameters dP/dt_max_ and dP/dt_min_ (Supplementary Fig. 3B,C).

To examine activation of the Wnt pathway in this model, we measured the level of active (non-phosphorylated) β-catenin. After TAC, active β-catenin was significantly lower in *PMCA4*^*−/−*^–TAC mice (Fig. 3f,g). Consistent with this finding, *TCF4*, a β-catenin target gene, was significantly reduced in *PMCA4*^*−/−*^–TAC compared with WT–TAC mice, as measured by qRT–PCR (Supplementary Fig. 3D). These results suggested that the Wnt/β-catenin pathway was downregulated after TAC in *PMCA4*^*−/−*^ mice compared with WT.

Next, we analysed how *PMCA4*^*−/−*^ mice responded to exercise-induced cardiac hypertrophy by subjecting them to swimming for 4 weeks. In contrast to the TAC model, the hypertrophic response to swimming exercise was unaltered (Supplementary Fig. 3E) suggesting that PMCA4 specifically regulates pathological but not physiological cardiac hypertrophy.

To analyse the extent of cardiac fibrosis, we stained heart tissue sections with Masson's trichrome staining. As shown in Supplementary Fig. 3F,G, we did not find any significant difference in the level of fibrosis between *PMCA4*^*−/−*^ mice and WT controls.

### *Pmca4* ablation protects against long-term pressure overload

Although our 5 weeks TAC model was sufficient to induce hypertrophy, we did not observe any significant effect on cardiac function as indicated by haemodynamic data. Therefore, we performed a more severe model by inducing long-term pressure overload for 12 weeks in *PMCA4*^*−/−*^ mice. With this TAC model, we found that WT mice had a dramatically reduced survival rate (40% survival after 12 weeks of TAC), while all of the *PMCA4*^*−/−*^ mice survived until 12 weeks following TAC (Fig. 3h). When we assessed cardiac function in surviving animals at 12 weeks after TAC, we found that *PMCA4*^*−/−*^ mice had significantly better contractility as indicated by dP/dt_max_ and dP/dt_min_ values (Supplementary Fig. 4A,B). These data showed that *Pmca4* gene deletion protected against the development of contractile dysfunction and improved the survival after long-term TAC.

### sFRP2 contributes to attenuated hypertrophy in *PMCA4* ^*−/−*^ mice

To investigate whether the protection against TAC-induced hypertrophy in *PMCA4*^*−/−*^ mice was due to the expression of sFRP2, we subjected *PMCA4*^*−/−*^ mice to TAC surgery and then treated them with anti-sFRP2 antibody (200 μg per kg body weight per day, intraperitoneally (i.p.)) or normal goat IgG at a similar dose as control. Immunoprecipitation analysis showed that injection with anti-sFRP2 antibody increased the interaction between Wnt and its receptor, Frizzled, in the heart (Supplementary Fig. 5A). This data indicated that anti-sFRP2 antibody treatment successfully blocked sFRP2 activity *in vivo*. Furthermore, we found that the hypertrophic response to TAC was restored in *PMCA4*^−/−^ mice treated with anti-sFRP2 antibody as indicated by analysis of left ventricular mass/TL ratio, cardiomyocyte cross-sectional area and BNP expression (Supplementary Fig. 5B–F). In contrast, *PMCA4*^*−/−*^ mice treated with control IgG displayed an attenuated hypertrophic response. The level of active β-catenin was remarkably increased following anti-sFRP2 treatment in TAC-treated mice, but in sham-treated animals we observed only slight (∼12%) elevation of active β-catenin level after anti-sFRP2 treatment (Supplementary Fig. 5G,H). This might be due to the fact that the Wnt pathway is basally inactive in the adult heart and re-activated in pathological conditions including pressure overload^18^. Overall, our data suggests that the anti-hypertrophic effect of PMCA4 ablation in mice was at least in part due to sFRP2 expression.

### Hypertrophy unaltered by *Pmca4* ablation in cardiomyocytes

**Figure 4:**
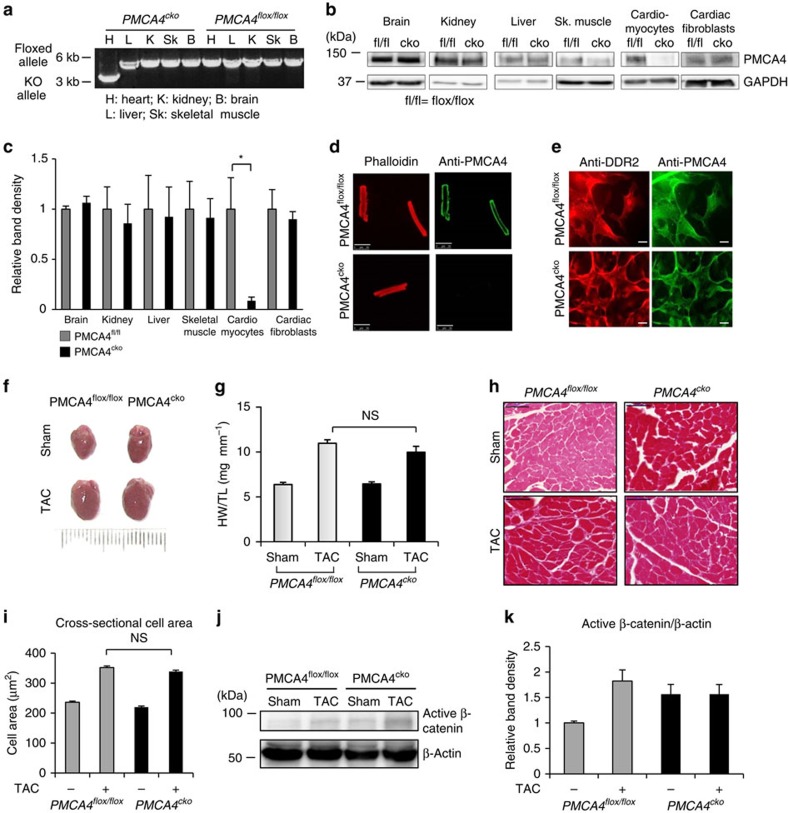
*Pmca4* deletion in cardiomyocytes did not affect TAC-induced hypertrophy. (**a**) PCR analysis confirming specific deletion of the *Pmca4* allele in the heart of *PMCA4*^*cko*^ mice. (**b**) Western blot analysis showing PMCA4 expression in several organs and in isolated cardiomyocytes and cardiac fibroblasts. (**c**) Quantification of band density (relative to expression in control mice) showed that expression of PMCA4 was ablated only in cardiomyocytes of *PMCA4*^*cko*^ mice (*n*=3; *P<0.05, Student's *t*-test). (**d**) Immunofluorescence experiment to detect PMCA4 expression in isolated adult cardiomyocytes and (**e**) in ACFs (scale bars, 50 μm). Cells were stained with anti-PMCA4 (green) and phalloidin or anti-DDR2 (red). The results showed absence of PMCA4 in cardiac myocytes but not in cardiac fibroblasts of *PMCA4*^*cko*^. (**f**) Images of hearts from *PMCA4*^*cko*^ and controls after 5 weeks of TAC stimulation. (**g**) HW/TL ratio indicated that there was no difference in hypertrophy between *PMCA4*^*cko*^ and control littermates (*PMCA4*^*flox/flox*^) after TAC (control sham, *n*=7; control TAC, *n*=9; *PMCA4*^*cko*^ sham, *n*=6; *PMCA4*^*cko*^ TAC, *n*=7, *P*=NS, one-way analysis of variance (ANOVA)). (**h**) Haematoxylin and eosin staining of histological sections and (**i**) analysis of cross-sectional cardiomyocyte area supported the finding of no difference in hypertrophic response between *PMCA4*^*cko*^ and controls (scale bars, 50 μM; *n*=4 per group). (**j**) Representative western blotting and (**k**) measurement of band density showed no difference in the level of active β-catenin between *PMCA4*^*cko*^ and controls (*n*=5 per group), NS, not significant. All error bars represent the s.e.m.

To investigate the effects of PMCA4 cell-specific ablation in the heart, we generated conditional KO models. We generated mice carrying *loxP* sequences flanking exons 2 and 3 of the *Pmca4* gene, which contain the start codon (Supplementary Fig. 6). We crossed these mice with αMHC-Cre transgenic mice to establish PMCA4 cardiomyocyte-specific KO mice (*PMCA4*^*cko*^). Expression analyses confirmed the specific ablation of PMCA4 in the cardiomyocytes, whereas the cardiac fibroblasts retained PMCA4 expression (Fig. 4a–e). We then subjected these mice to TAC for 5 weeks and used *Pmca4* floxed mice (*PMCA4*^*flox/flox*^) as controls. After 5 weeks of pressure overload, *PMCA4*^*cko*^ did not display differences in the hypertrophic response, as indicated by HW/TL ratio, cardiomyocyte cross-sectional area and echocardiographic analysis (Fig. 4f–i and Supplementary Table 2). In addition, the level of ANP expression was not different between *PMCA4*^*cko*^ and *PMCA4*^*flox/flox*^ mice (Supplementary Fig. 7A). By analysing cardiac haemodynamics (dP/dt_max_ and dP/dt_min_), we also found that there was no difference in cardiac contractile function between *PMCA4*^*flox/flox*^ and *PMCA4*^*cko*^ mice after TAC (Supplementary Fig. 7B,C). Furthermore, active β-catenin levels and *TCF4* gene expression were not different between *PMCA4*^*cko*^ and *PMCA4*^*flox/flox*^ mice after TAC, suggesting that Wnt/β-catenin signal activation was unaltered (Fig. 4j,k and Supplementary Fig. 7D). Similar to the data from *PMCA4*^*−/−*^ mice, *PMCA4*^*cko*^ did not show any difference in the level of fibrosis compared with controls (Supplementary Fig. 7E,F). Together, this data confirmed that the protective effect of PMCA4 ablation was not through signalling in cardiomyocytes.

### *Pmca4* deletion in fibroblasts reduces hypertrophy after TAC

**Figure 5:**
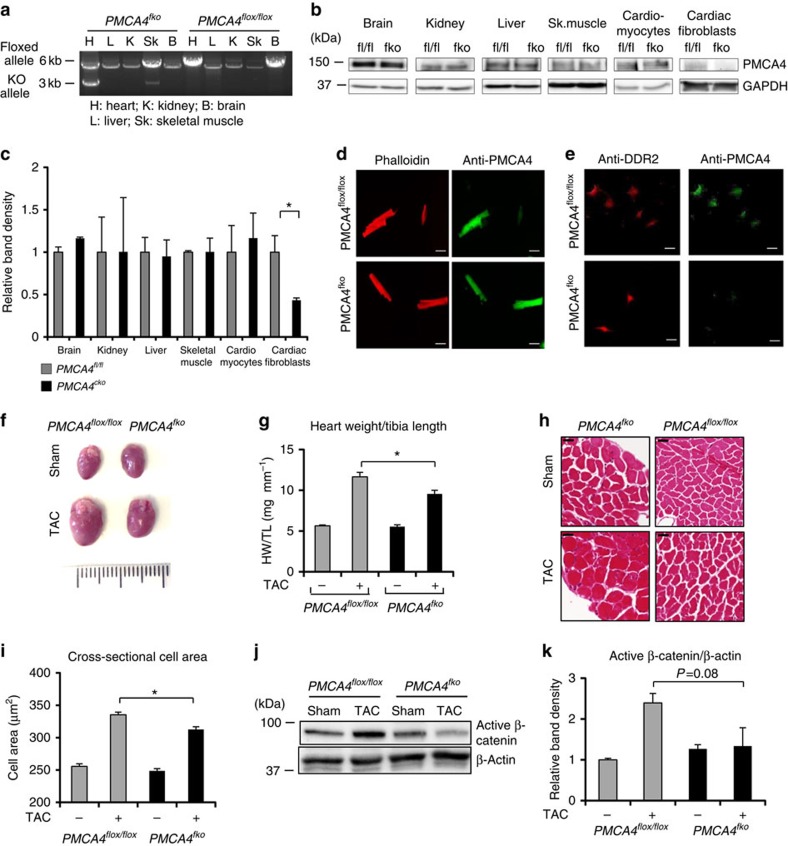
Fibroblast-specific *Pmca4* ablation reduced TAC-induced hypertrophy. (**a**) Image of PCR analysis to detect the deleted *Pmca4* allele in *PMCA4*^*fko*^ mice. (**b**) Western blotting showing the expression of PMCA4 in several different organs and in isolated cardiomyocytes and cardiac fibroblasts. (**c**) Quantification of band density (relative to expression in control mice) showed that expression of PMCA4 was ablated only in cardiac fibroblasts of *PMCA4*^*fko*^ mice (*n*=3; *P<0.05, Student's *t*-test). (**d**) Adult cardiomyocytes and (**e**) cardiac fibroblasts were stained with anti-PMCA4 (green) and phalloidin or anti-DDR2 (red). Expression of PMCA4 was ablated in isolated ACFs but not in cardiomyocytes of *PMCA4*^*fko*^ mice (scale bars, 25 μm). (**f**) Image of hearts from *PMCA4*^*fko*^ and control littermates after TAC for 5 weeks. (**g**) HW/TL ratio analysis showed significantly reduced hypertrophy in *PMCA4*^*fko*^ mice following TAC (control sham, *n*=4; control TAC, *n*=5; *PMCA4*^*fko*^ sham, *n*=4; *PMCA4*^*fko*^ TAC, *n*=4, *P<0.05, one-way analysis of variance (ANOVA)). (**h**) Examples of histological sections stained with haematoxylin and eosin, and (**i**) calculation of cross-sectional cardiomyocyte area indicated a significantly reduced hypertrophy in *PMCA4*^*fko*^ mice (scale bars, 50 μm, control sham, *n*=4; control TAC, *n*=5; *PMCA4*^*fko*^ sham, *n*=4; *PMCA4*^*fko*^ TAC, *n*=4; *P<0.05, one-way ANOVA). (**j**) Active β-catenin level was detected by western blotting. (**k**) Band density assessment suggested a strong trend (*P*=0.08) in reduced β-catenin activation in *PMCA4*^*fko*^ following TAC (control sham, *n*=4; control TAC, *n*=5; *PMCA4*^*fko*^ sham, *n*=4; *PMCA4*^*fko*^ TAC, *n*=4). All error bars represent the s.e.m.

Next, we generated a PMCA4 fibroblasts-specific KO (*PMCA4*^*fko*^) by crossing *PMCA4*^*flox/flox*^ mice with *Postn*-Cre transgenic mice. These mice express Cre recombinase under the control of the Periostin (*Postn*) gene promoter^8^,^22^ and have been demonstrated to knock out genes specifically in cardiac fibroblasts but not in cardiomyocytes^8^,^23^. The deleted allele was present in DNA isolated from the heart of *PMCA4*^*fko*^, although as expected, the WT (non-deleted) allele was also present in the heart, presumably as a consequence of DNA from cardiomyocytes (Fig. 5a). Using western blotting and immunofluorescence, we found specific ablation of PMCA4 expression in isolated cardiac fibroblasts of *PMCA4*^*fko*^ mice, whereas expression of PMCA4 in cardiomyocytes remained unaltered (Fig. 5b–e). To induce pressure-overload hypertrophy, we subjected *PMCA4*^*fko*^ to TAC for 5 weeks. In contrast to the data from *PMCA4*^*cko*^ mice, *PMCA4*^*fko*^ exhibited a significantly reduced hypertrophic response, as indicated by measurement of HW/TL ratio, the cross-sectional area of cardiomyocytes and analysis of echocardiographic parameters (Fig. 5f–i and Supplementary Table 3). Furthermore, qRT–PCR data showed a significantly reduced level of the hypertrophic marker ANP in *PMCA4*^*fko*^ after TAC (Supplementary Fig. 8A). However, consistent with data from *PMCA4*^*−/−*^ mice, contractile function was unchanged in *PMCA4*^*fko*^ compared with control mice following TAC (Supplementary Fig. 8B,C). By analysing Wnt/β-catenin signal activation, we found that both active β-catenin and expression of *TCF4* were decreased in *PMCA4*^*fko*^ mice after TAC compared with WT (Fig. 5j,k and Supplementary Fig. 8D). However, consistent with data obtained from *PMCA4*^*−/−*^ mice there was no difference in the level of fibrosis in *PMCA4*^*fko*^ compared with control mice (Supplementary Fig. 8E,F). These results clearly supported the idea that signal modulation by PMCA4 in cardiac fibroblasts was important in mediating the anti-hypertrophic effect.

### Pharmacological PMCA4 inhibition enhances sFRP2 expression

Data from our animal models together with observations in isolated cardiac fibroblasts prompted us to speculate that a reduction in PMCA4 activity in cardiac fibroblasts will provide a beneficial effect against pathological stimuli in the heart. By screening a library of medically optimized drug-like molecules we have identified aurintricarboxylic acid (ATA) as a potent pharmacological inhibitor of PMCA4 (ref. 24). ATA has an IC50 of 150 nM for PMCA4 inhibition and only produced a minor effect on the second isoform of PMCA expressed in the heart, PMCA1 (ref. 24). We found that ATA inhibits PMCA4 activity at a very low concentration (IC50=150 nM) (ref. 24). However, it is also known that at higher concentrations ATA also inhibits other enzymes, such as nucleases (at 10–50 μM) (ref. 25), calpain (IC50=22 μM) (ref. 26) and influenza virus neuraminidase (at 100 μg ml^−1^ or ∼210 μM) (ref. 27). Therefore, in this study we used a very low dose of ATA (1 μM), to ensure that its effects are probably only due to PMCA4 inhibition.

**Figure 6:**
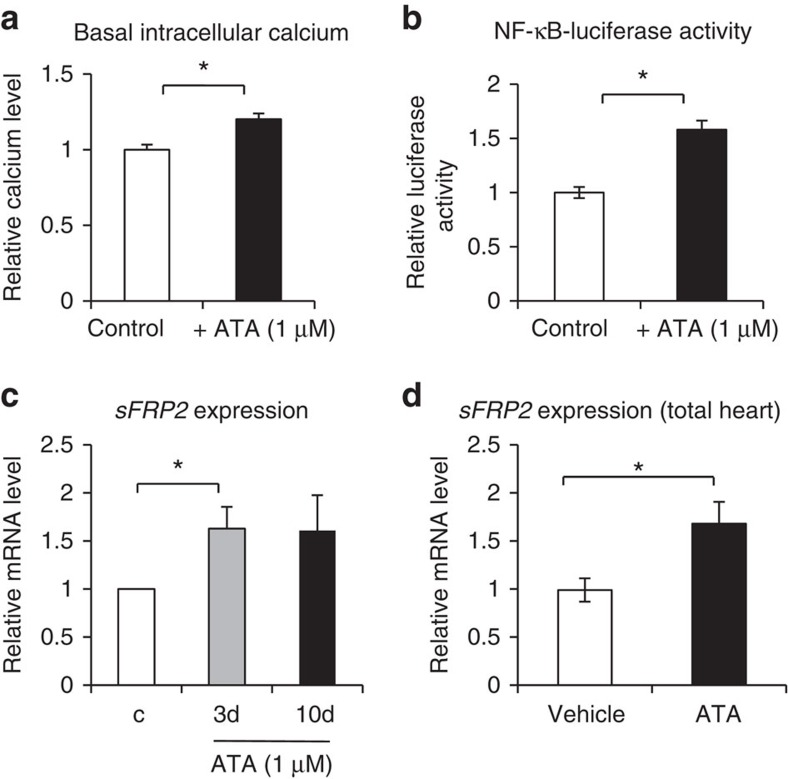
PMCA4 inhibition increased sFRP2 levels in cardiac fibroblasts and the heart. (**a**) ACFs isolated from WT mice were subjected to ATA treatment (1 μM for 48 h). Measurement of basal intracellular calcium using fluo-3 dye indicated a significantly higher calcium level in ATA-treated cells (*n*=4; *P<0.05, Student's *t*-test). (**b**) Analysis of NF-κB activity using the NF-κB-luciferase construct showed a significant increase in NF-κB activity in ATA-treated cells (*n*=4; *P<0.05, Student's *t*-test). (**c**) ACFs were treated with ATA (1 μM) for 3 or 10 days. qRT–PCR analysis showed that sFRP2 level was significantly enhanced in ATA-treated cells (*n*=4; *P<0.05, Student's *t*-test). (**d**) WT mice were injected with ATA (5 mg kg^−1^ per day for 2 weeks). sFRP2 level was detected in total heart's mRNA. Results showed a significant increase in sFRP2 level (*n*=6–8 in each group; *P<0.05, Student's *t*-test). All error bars represent the s.e.m.

We treated WT cardiac fibroblasts with ATA at this dose for 48 h and found that it increased the basal level of calcium (Fig. 6a) and NF-κB activity (Fig. 6b). We also found that extending this treatment to 3 or 10 days significantly enhanced sFRP2 expression in isolated WT cardiac fibroblasts (Fig. 6c). To examine whether ATA treatment increased cardiac sFRP2 expression *in vivo*, we injected WT C57Bl/6 mice with ATA (5 mg per kg body weight per day, i.p.) for 2 weeks. We found that cardiac sFRP2 was significantly increased in ATA-treated mice (Fig. 6d).

### ATA treatment attenuates TAC-induced hypertrophy

**Figure 7:**
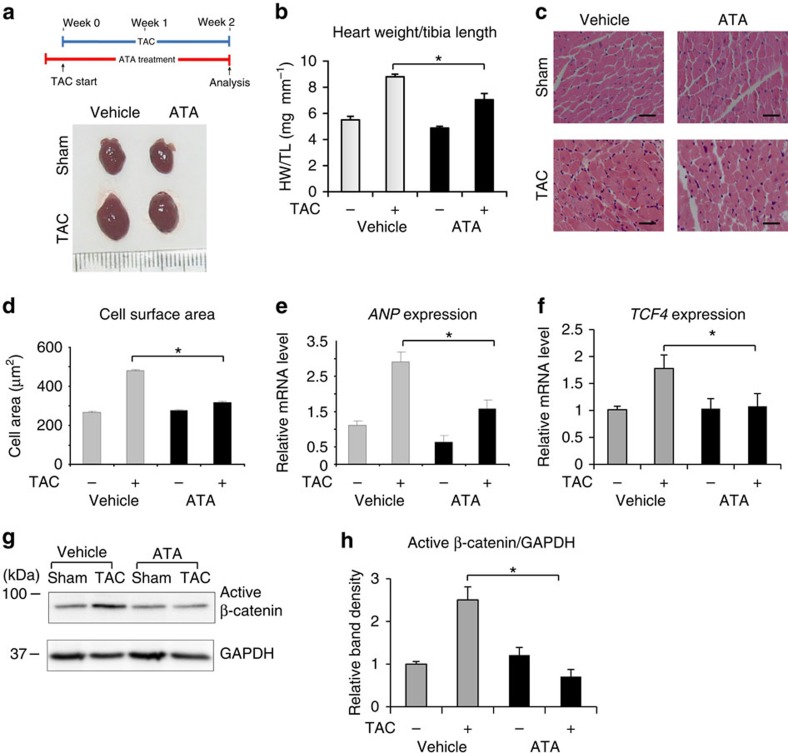
ATA treatment reduced cardiac hypertrophy in response to pressure overload. (**a**) Schematic diagram of treatment strategy and gross morphology of mouse hearts following TAC (2 weeks) with or without ATA treatment (5 mg kg^−1^ per day, i.p.). (**b**) Analysis of HW/TL ratio showed significantly reduced hypertrophy in mice treated with ATA (sham vehicle, *n*=9; TAC vehicle, *n*=11; sham ATA, *n*=8; TAC ATA, *n*=9; *P<0.05, one-way analysis of variance (ANOVA) followed by *post-hoc* multiple comparison test) (**c**) Haematoxylin and eosin staining of histological sections and measurement of cross-sectional cell surface area (scale bars, 50 μm) (**d**) indicated that ATA treatment significantly diminished the hypertrophic response (sham vehicle, *n*=9; TAC vehicle, *n*=11; sham ATA, *n*=8; TAC ATA, *n*=9; *P<0.05, one-way ANOVA followed by *post-hoc* multiple comparison test). (**e**) Expression of ANP and (**f**) *TCF4* were detected by real-time RT–PCR. ATA-treated mice showed significantly lower expression of both ANP and *TCF4* (sham vehicle, *n*=3; TAC vehicle, *n*=6; sham ATA, *n*=3; TAC ATA, *n*=4; *P<0.05). (**g**) Western blotting of active β-catenin and (**h**) quantification of band density indicated a significant reduction of active β-catenin level in TAC-operated animals treated with ATA (*P<0.05, one-way ANOVA followed by *post-hoc* multiple comparison test). All error bars represent the s.e.m.

Next, we tested the effects of ATA on pressure-overload hypertrophy *in vivo*. We treated WT C57Bl/6 mice with ATA (5 mg per kg body weight per day) from day 3 before TAC until 2 weeks after. ATA remarkably reduced the hypertrophic response as indicated by decreased HW/TL ratio, cell surface area and expression of the hypertrophic marker ANP (Fig. 7a–e). To analyse activation of the Wnt/β-catenin pathway, we examined the levels of *TCF4* gene expression and active β-catenin in the heart, and found that they were downregulated in TAC animals treated with ATA (Fig. 7f–h). However, cardiac contractility was unaltered (Supplementary Fig. 9A,B), which was consistent with the data from our KO models.

### ATA reverses established cardiac hypertrophy

**Figure 8:**
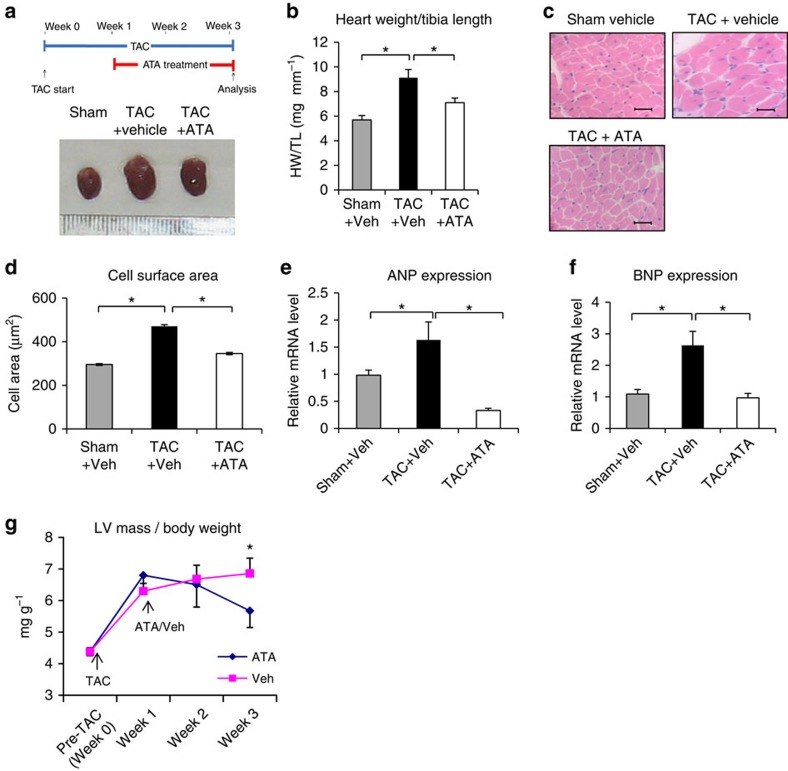
ATA reversed already established cardiac hypertrophy in WT mice. (**a**) Schematic diagram of treatment strategy and gross morphology of hearts at the end of experiments. (**b**) Analysis of HW/TL ratio showed that ATA treatment (5 mg kg^−1^ per day, i.p.) significantly reduced established cardiac hypertrophy (sham vehicle, *n*=4; TAC vehicle, *n*=4; TAC ATA, *n*=6; *P<0.05) (**c**) Examples of histological sections (scale bars, 50 μm). (**d**) Analysis of cell surface area measurement supported the HW/TL ratio data (sham vehicle, *n*=4; TAC vehicle, *n*=4; TAC ATA, *n*=6; *P<0.05). (**e**) Expression of hypertrophic markers ANP and (**f**) BNP after 2 weeks of ATA treatment supported the finding that ATA reversed established hypertrophy in mice. (**g**) Serial echocardiography analysis showed that the left ventricular mass was remarkably increased 1 week after TAC and the reduction in left ventricular mass occurred following the treatment with ATA (*P<0.05, ATA- versus vehicle-treated group). All error bars represent the s.e.m.

We then tested whether ATA could reverse previously established cardiac hypertrophy (‘treatment strategy') (Fig. 8a). Starting 1 week after TAC, we treated C57Bl/6 mice with ATA at 5 mg per kg body weight per day. Echocardiography confirmed myocardial hypertrophy at this stage (Supplementary Fig. 10A). Histological analysis, HW/TL ratio measurement and expression of hypertrophic markers ANP and BNP showed that ATA treatment significantly reversed the hypertrophic response (Fig. 8b–f). Furthermore, serial echocardiography analysis revealed that the reduction in left ventricular mass/TL ratio began after ATA treatment (Fig. 8g), suggesting that the effect was due to ATA treatment. Similar to our other models, we did not observe changes in cardiac contractility in this model (Supplementary Fig. 10B,C).

As an initial step to assess whether systemic treatment of ATA (5 mg per kg body weight per day for 2 weeks) produces adverse effects in other major organs such as the liver and kidney, we measured the level of serum alanine transaminase, aspartate transaminase and creatinine in ATA-treated mice. As shown in Supplementary Fig. 11A,B, we did not find any difference between ATA- and vehicle-treated mice. This suggested that the ATA treatment was unlikely to cause toxicity in major organs such as the liver and kidney.

### ATA treatment in *PMCA4* knockout mouse models

**Figure 9:**
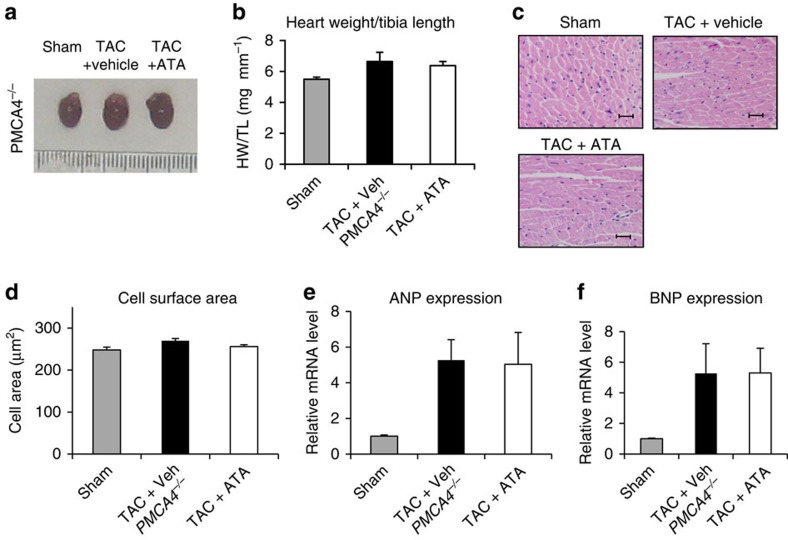
ATA had no additional effect on hypertrophy in *PMCA4*^*−/−*^ mice following TAC. (**a**) Image of *PMCA4*^*−/−*^ hearts at the end of experiments. (**b**) Measurement of HW/TL ratio and analysis of cross-sectional cell surface area on histological sections (scale bars, 50 μm). (**c**,**d**) ATA treatment did not affect hypertrophic response in *PMCA4*^*−/−*^ mice (*n*=4–6 in each group). (**e**) The level of ANP and (**f**) BNP were not altered by ATA treatment in *PMCA4*^*−/−*^ mice. All error bars represent the s.e.m.

To further confirm that the anti-hypertrophic effect of ATA is due to the inhibition of PMCA4, we performed TAC on PMCA4 global KO mice (*PMCA4*^*−/−*^) and treated them with ATA. If ATA had additional PMCA4-independent effects, the expectation would be that it would further reduce HW, cell size and expression of hypertrophic markers in *PMCA4*^*−/−*^ animals. Data shown in Fig. 9a–f showed that ATA treatment did not further reduce these parameters in the *PMCA4*^*−/−*^ mice.

**Figure 10:**
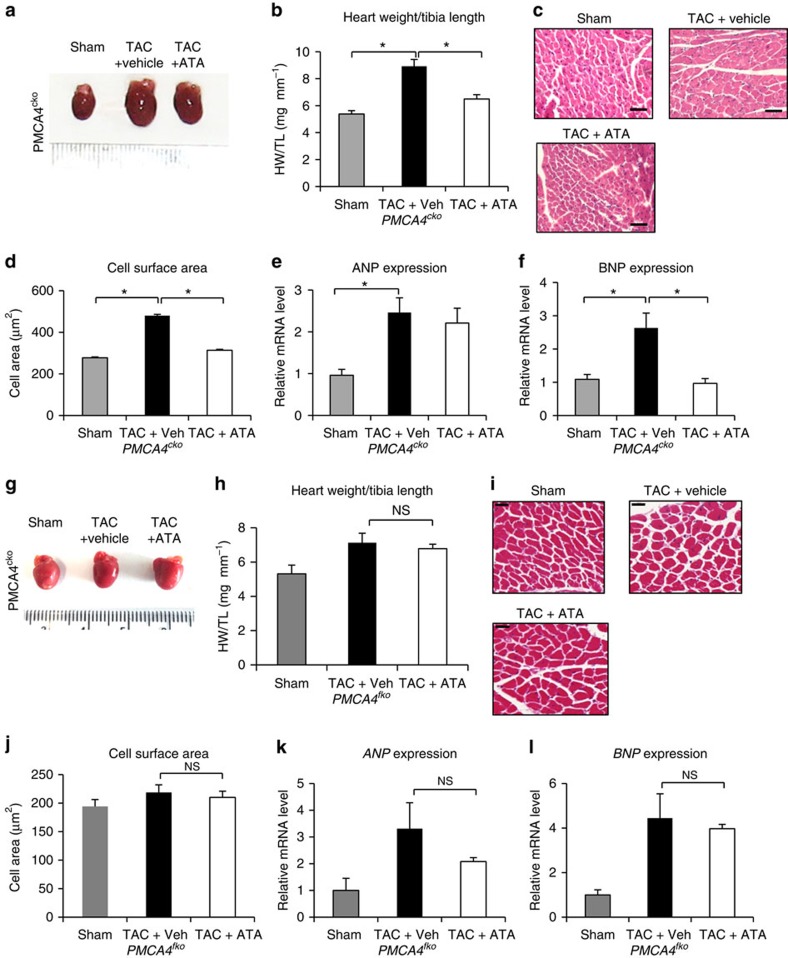
ATA inhibited cardiac hypertrophy in *PMCA4*^*cko*^ but not *PMCA4*^*fko*^ mice. (**a**) Gross morphology of the hearts from *PMCA4*^*cko*^ mice at the end of the experiments. (**b**) HW/TL ratio measurement indicated significantly reduced hypertrophy after ATA treatment in *PMCA4*^*cko*^ mice (sham vehicle, *n*=5; TAC vehicle, *n*=6; TAC ATA, *n*=6; *P<0.05, one-way analysis of variance (ANOVA)). (**c**) Representative histological sections (scale bars, 50 μm) and (**d**) measurement of cell surface area showed a significant reduction of hypertrophy following ATA treatment (sham vehicle, *n*=5; TAC vehicle, *n*=6; TAC ATA, *n*=6; *P<0.05, one-way ANOVA). (**e**) ANP level did not differ following ATA treatment but BNP expression (**f**) was significantly reduced in *PMCA4*^*cko*^-TAC after ATA treatment (sham vehicle, *n*=5; TAC vehicle, *n*=5; TAC ATA, *n*=4; *P<0.05). (**g**) Hearts from *PMCA4*^*fko*^ following TAC with or without ATA treatment. (**h**) Analysis of HW/TL ratio and (**i**,**j**) cross-sectional cardiomyocyte area (scale bars, 50 μm) showed that ATA treatment did not alter the hypertrophic response in *PMCA4*^*fko*^ mice (sham vehicle, *n*=5; TAC vehicle, *n*=4; TAC ATA, *n*=4). (**k**) The level of ANP and (**l**) BNP expression were not significantly different between TAC+vehicle and TAC+ATA group (NS, not significant). All error bars represent the s.e.m.

We then examined whether ATA had any anti-hypertrophic effects when PMCA4 was specifically ablated in cardiomyocytes. As shown in Fig. 10a–f, ATA treatment reduced hypertrophy in *PMCA4*^*cko*^ mice as indicated by HW/TL ratio, cell size measurement and analysis of the hypertrophic marker BNP, suggesting that the anti-hypertrophic effect of ATA *in vivo* was not via modulating PMCA4 in cardiomyocytes. To confirm this finding *in vitro*, we tested the effect of ATA treatment on isolated cardiomyocytes. At 1 μM, ATA did not significantly alter phenylephrine-induced cardiomyocyte hypertrophy (30 μM phenylephrine for 72 h), as indicated by cell surface area analysis (Supplementary Fig. 12A,B).

Finally, we evaluated the effects of ATA treatment on *PMCA4*^*fko*^ mice. In contrast to the data obtained from *PMCA4*^*cko*^ mice, ATA treatment did not produce an anti-hypertrophic effect in *PMCA4*^*fko*^ mice as shown in Fig. 10g–l. Together, the data provide strong support that the anti-hypertrophic effect of ATA was due to modulation of PMCA4 in cardiac fibroblasts.

## Discussion

Overall, our results show that PMCA4 is a key regulator of pathological cardiac hypertrophy that can be pharmacologically targeted. With global and conditional KO models and an *in vitro* system, we demonstrated that PMCA4 mediates a paracrine mechanism in the heart. Inhibiting PMCA4, both genetically and pharmacologically, elevated expression of sFRP2 in cardiac fibroblasts, which inhibited Wnt signalling in cardiomyocytes and protected them from developing pathological hypertrophy. Importantly, we also showed that a novel and potent inhibitor of PMCA4, ATA^24^, efficiently reduced pathological hypertrophy. These data provide *in vivo* evidence that PMCA4 is a promising and readily ‘druggable' target for anti-hypertrophic therapy.

The role of cardiac fibroblasts in mediating cellular signalling in the heart is increasingly evident. Traditionally, fibroblasts have been regarded as the main regulator of extracellular matrix composition due to their capacity in producing components of the extracellular matrix^28^. However, recent knowledge has suggested that cardiac fibroblasts can mediate signal transmission in the heart through paracrine mechanisms or cell–cell contact^7^. Cardiac fibroblasts secrete a number of proteins that regulate key processes in the heart, such as the pro-hypertrophic factor fibroblast growth factor-2 (ref. 29), the cytoprotective substance IGF1 (ref. 8), and interleukin-33, which are also anti-hypertrophic^30^. Furthermore, cardiac fibroblasts may also release microRNA to the neighbouring cells, including miR21-3p, a fibroblast-derived microRNA, which modulates cardiac hypertrophy^9^.

On the basis of this knowledge, we studied calcium signalling in cardiac fibroblasts, in particular those regulated by PMCA4. This is because PMCA4 is known to be a major regulator of local calcium signals in cardiomyocytes^21^,^31^,^32^,^33^, but its role in cardiac fibroblasts is relatively unknown. Our observations demonstrate that PMCA4 regulates expression of major signalling molecules in cardiac fibroblasts. Specifically, we identified that inhibiting PMCA4 expression/activity significantly increased expression of sFRP2, a potent inhibitor of the Wnt pathway. The mechanism may be due to the increase in NF-κB activity and *Pax2* expression level. *Pax2* is the major transcription factor responsible for sFRP2 expression^14^, whereas NF-κB regulates *Pax2* expression^15^. Consistent with this, we showed that *Pax2* expression and NF-κB activity were increased in *PMCA4*^*−/−*^ fibroblasts. Increased NF-κB activity might be caused by increased resting levels of intracellular calcium in *PMCA4*^*−/−*^ fibroblasts, similar to the mechanism described previously in mdx skeletal myotubes^17^.

The Wnt signalling pathway is a major regulator of cardiac development, hypertrophy and failure; it is normally active during embryonic development and is re-activated under pathological conditions^18^. Activation of this pathway will induce the canonical downstream pathway via β-catenin. The overall result is the activation of a gene programme for growth and development^18^,^34^. Consistent with this knowledge, our *PMCA4*^*−/−*^ mice, which express higher levels of Wnt inhibitor sFRP2, exhibited a reduced hypertrophic response to pressure-overload stimulus. Experiments using conditional KO models together with analyses using a cellular model strongly indicate a paracrine mechanism in this process. In addition, treatment with anti-sFRP2 antibody abolishes the anti-hypertrophic effect of PMCA4 ablation in mice providing direct *in vivo* evidence that sFRP2 is a downstream effector of PMCA4-mediated signalling.

In these models we did not observe any alteration in cardiac function following 5 weeks of TAC in both WT and KO mice. However, we found a dramatic reduction in heart function and a higher mortality rate in WT mice compared with *PMCA4*^*−/−*^ mice after 12 weeks of TAC, indicating that PMCA4 ablation may preserve cardiac contractile function and protect against the development of heart failure in the chronic pressure overload model.

Despite protection against pressure-overload-induced enlargement of the cardiomyocytes, we did not observe a significant reduction in cardiac fibrosis in our global and cell-specific PMCA4 KO strains following TAC surgery. Previous studies have provided conflicting evidence regarding the role of sFRP2 in mediating cardiac fibrosis in the myocardial infarction model. Kobayashi *et al*.^13^ showed that genetic ablation of sFRP2 reduced the extent of cardiac fibrosis following myocardial infarction, suggesting that sFRP2 might promote the development of fibrosis. In contrast, other studies have demonstrated that injection of recombinant sFRP2 directly into an infarcted heart resulted in attenuated remodelling and fibrosis^12^, indicating a protective role for sFRP2. It is well observed that in general the extent of fibrosis following TAC is considerably less than after myocardial infarction and, as such, this may explain why we did not see any difference in the fibrosis level in our models. It would therefore be very interesting to evaluate the effect of PMCA4 inhibition in a model of myocardial infarction; however, these experiments are beyond the scope of this study.

The process of generating our PMCA4 global KO mice has been described previously^20^. We targeted exon 2 and part of exon 3 of the mouse *Pmca4* gene using a neomycin cassette. As exon 2 contains the start codon, this strategy completely ablated PMCA4 protein from the heart, as described in our previous publication^21^. Another study, described after that publication, developed a second *Pmca4*-targeted mouse line, in which codons 448–474 in exon 11 were replaced by a neomycin cassette^35^. This strategy did not result in a complete KO, as the truncated *Pmca4* mRNA transcript could still be detected in all tissues tested^35^. Furthermore, Afroze *et al*.^36^ compared the expression of PMCA4 in both KO lines and demonstrated that PMCA4 was completely absent from our KO mice, but not the other line. The difference in the nature of the KO models may explain the discrepancy of the phenotypes observed in these two lines of KO mice. Preliminary descriptive data by Wu *et al*.^33^ suggested a minimal (<10%) increase in the hypertrophic response to TAC in the ‘exon 11 targeted' PMCA4 KO line. However, further study using this KO line is in agreement with our current data: it was shown that cross-breeding of a hypertrophic cardiomyopathy mouse model with this PMCA4 KO line resulted in the reduction of hypertrophy^37^. In addition, our recent findings are supported by data from two other conditional KOs, which should clarify that any discrepancy is due to expression level as described above.

It is also important to note that PMCA4 may also have a signalling role in the cardiomyocytes. We previously showed that moderately overexpressing PMCA4 (∼2-fold) in cardiac myocytes reduced the β-adrenergic contractile response and exaggerated the hypertrophic response following chronic stimulation with isoproterenol^32^. In contrast, overexpressing PMCA4 at high levels (∼6-fold) in cardiomyocytes reduced hypertrophy following TAC^33^. In the latter model, PMCA4 recruited calcineurin, which eventually reduced the calcineurin/nuclear factor of activated T-cells (NFAT) signal activity^33^. Thus, overexpressing PMCA4 at non-physiological levels may produce an anchoring effect to its interacting partners, including calcineurin. Indeed, in cardiomyocytes isolated from our *PMCA4*^*−/−*^ mice, membrane calcineurin was reduced but total calcineurin was unaltered (Supplementary Fig. 13). In support of this notion, we recently showed that PMCA4 exerts an anchoring effect on neuronal nitric oxide synthase (nNOS)^21^. This suggests that the effects of PMCA4 overexpression might depend on the level of transgene expression and might explain the phenotype discrepancy between different transgenic lines.

Although systemic ablation of PMCA4 resulted in almost complete attenuation of the hypertrophic response, the effect of PMCA4 KO in fibroblasts showed a significant but not complete attenuation of the hypertrophic response. This indicated that PMCA4 ablation in other cell types such as endothelial and smooth muscle cells might contribute to the modulation of cardiac hypertrophy. Indeed, several reports have shown that PMCA4 regulates important signalling pathways in endothelial cells (that is, the vascular endothelial growth factor-mediated angiogenesis)^38^ and in smooth muscle cells (that is, the neuronal nitric oxide synthase (nNOS)-mediated contraction)^39^. Further studies are needed to fully characterize the role of PMCA4 in endothelial cells and smooth muscle cells in the regulation of cardiac hypertrophy.

Perhaps the most important aspect of this study is the possible translational implications. We have demonstrated in this study that interfering with the PMCA4-mediated signal using a pharmacological inhibitor may be beneficial for controlling pathological hypertrophy. The nature of PMCA4 as a membrane protein makes this molecule pharmacologically targetable. In addition, ATPases are highly amenable to large-scale chemical library screens. Using a medium-scale screening system we have identified ATA as a potent PMCA4 inhibitor^24^. Strikingly, inhibition of PMCA4 using ATA could suppress and reverse cardiac hypertrophy. Observations using global and conditional KO mouse models, as well as *in vitro* experiments by treating isolated cardiomyocytes with ATA (1 μM), strongly support the idea that ATA mainly works in cardiac fibroblasts but not in myocytes. Thus, this study provides direct evidence that cardiac fibroblasts can be targeted pharmacologically to treat myocardial hypertrophy.

Whole-body phenotyping of our *PMCA4*^*−/−*^ mice, including analyses of blood chemistry, nervous system, X-ray and dexascan (see Supplementary Table 4) revealed no abnormalities, indicating that targeting this molecule is unlikely to cause side effects in any of these organ systems. However, our previous observations showed that male *PMCA4*^*−/−*^ mice exhibited reduced sperm motility^20^. Although the effect of systemic administration of a PMCA4 inhibitor on fertility needs addressing, it should be noted that there is a blood–testis barrier similar to the blood–brain barrier that most small molecules do not cross^40^.

Although industrially refined derivatives may be needed for human drug development, ATA provides proof of principle for our approach. Not only did it prevent pathological hypertrophy when applied before overload but it also considerably reduced hypertrophy when applied after the establishment of hypertrophy, which is a more realistic scenario for translating these results to future clinical studies.

## Methods

### Animals

Mice with a targeted deletion of exon 2 and part of exon 3, which contain the start codon of the *Pmca4* gene were used in this study^20^. We used 8- to 10-week-old male PMCA4 KO mice for pressure-overload or exercise-induced hypertrophy experiments. Age- and sex-matched WT littermates were used as controls. For tissue-specific KOs, we generated the *PMCA4*^*flox/flox*^ mice by flanking exon 2 and exon 3 of the *Pmca4* gene with *lox*P sites (Supplementary Fig. 6). To generate cardiomyocyte-specific KOs, we crossed these animals with αMHC-Cre mice (obtained from Dr Michael Schneider's laboratory^41^). To generate fibroblast-specific KO, we crossed *PMCA4*^*flox/flox*^ mice with *Postn*-Cre mice (a generous gift from Dr Simon Conway, Indiana University School of Medicine^22^). We used male, 8–10 weeks old C57Bl/6 mice, to test the effect of ATA during pressure overload hypertrophy. Animal studies were performed in accordance with the United Kingdom Animals (Scientific Procedures) Act 1986 and were approved by the University of Manchester Ethics Committee.

### Transverse aortic constriction

To produce a model of cardiac pressure overload, mice were subjected to TAC or a sham operation. Mice were induced with 5% isofluorane, orally intubated and then placed on a ventilator set to 200 breaths per minute, tidal volume 0.1 ml (Minivent 845, Harvard Apparatus). Anaesthesia was maintained at 3% isofluorane in 100% O_2_ throughout the surgery. With the aid of a binocular stereomicroscope (Olympus) the chest was opened via minithoracotomy, to expose the aortic arch, and TAC was performed by tying a 7–0 silk suture around a 27-gauge needle overlying the arch at the point between the brachiocephalic trunk and left common carotid artery. In our hands, this typically produces a ∼25–30 mm Hg pressure gradient between the left and right carotid artery^42^,^43^. For sham operations, the arch was exposed and a suture was passed around the back of the aorta before removal without tying. The chest was then sutured and 0.1 mg per kg body weight of buprenorphine was administered via i.p. injection. Mice were recovered in a 30 °C incubator before returning to normal housing. Heart tissues were collected at the end of experiments, for histology and molecular analyses.

For TAC experiments involving anti-sFRP2 antibody administration, 8-week-old *PMCA4*^*−/−*^mice were injected i.p. with either 200 μg per kg control goat IgG or goat anti-mouse sFRP2 antibody (R&D Systems) 1 day before TAC. They were then injected i.p. at this dose daily for a further 2 weeks (5 out of 7 days).

### Echocardiography and haemodynamic analyses

For haemodynamic analysis, mice were anaesthetised by i.p. injection of tribromoethanol (Avertin, 250 mg per kg body weight, Sigma). A midline cervical incision was made and the sternohyoid muscles were retracted, to expose the right carotid artery, which was tied at the bifurcation point, preventing regurgitation from the periphery. The right carotid artery was occluded proximally, allowing an incision to be made with minimal blood loss. A 1.4F pressure–volume catheter (SPR-839, Millar Instruments) was inserted via the carotid artery and ascending aorta into the left ventricle. Pressure–volume changes were recorded using a PowerLab system (Millar Instruments) once traces had stabilized. Inotropic and lusitropic function were assessed through analysis of the maximum and minimum rates of left ventricular pressure change, dP/dt_max_ and dP/dt_min_, respectively, using Millar's PVAN software.

Transthoracic echocardiography was conducted to monitor the progression of hypertrophy following TAC in the ATA treatment experiments under anaesthesia with 1.5% isofluorane. An Acuson Sequoia C256 ultrasound system fitted with a 14-MHz transducer (Siemens) was used to image the heart in the two-dimensional short-axis view, whereon M-mode echocardiography was recorded, to measure the left ventricular end diastolic diameter (LVEDD), left ventricular end systolic diameter, and diastolic posterior wall (dPW) and interventricular septal (dIVS) thicknesses in diastole. Measurements were obtained using the leading-edge method over a minimum period of three cardiac cycles, with the researcher blinded to mouse genotype and treatment. Mean wall thickness was determined by averaging the diastolic PW and IVS thicknesses, whereas LV mass was calculated using the formula 1.055 × [(LVEDD+dPW+dIVS)^3^−LVEDD^3^].

### Exercise-induced hypertrophy

To produce a model of exercise-induced hypertrophy, mice were swum twice a day for 90 min, 5 out of 7 days for a period of 4 weeks. An initial training period commenced exercise at 2 × 10 min per day, which was then increased in increments of 20 min per day until the full 2 × 90 min per day was reached. Following each swim, mice were towel dried and then maintained at 30 °C until fully dry, with 4 h rest between sessions. Water was maintained at a temperature of 30–32 °C in 18-l tanks at a depth of 30 cm. Sedentary controls were handled daily.

### Isolation of adult and neonatal cardiomyocytes

Adult mouse cardiomyocytes were isolated from 3- to 4-month-old animals. In brief, mice were killed by cervical dislocation, the hearts were rapidly removed and then perfused via the aorta with isolation solution pH 7.34 (134 mM NaCl, 11 mM glucose, 4 mM KCl, 1.2 mM MgSO_4_, 1.2 mM NaH_2_PO_4_ and 10 mM HEPES) for 4 min, followed by 9 min perfusion with a solution containing 0.6 and 0.075 mg ml^−1^ of collagenase type II (Worthington) and proteases type XIV (Sigma-Aldrich), respectively. Hearts were then perfused with Tyrode solution containing 50 mM taurine pH 7.34 for 12 min. The ventricles were cut from the heart and placed in Tyrode-taurine solution. The ventricles were then cut in half and pipetted up and down through a Pasteur pipette in 5 ml of Tyrode-taurine solution, to release the cardiomyocytes.

Adult rat cardiomyocytes were isolated and cultured following a protocol described by O'Connell *et al*.^44^ Adult female Sprague–Dawley rats were anaesthetized with 3% isoflurane and injected i.p. with 1,000 IU per kg body weight heparin. Hearts were then excised, cannulated via the aorta and Langendorff perfused with perfusion buffer (120.4 mM NaCl, 5.5 mM glucose, 14.7 mM KCl, 1.2 mM MgSO_4_-7H_2_O, 0.6 mM Na_2_HPO_4_, 10 mM Na-HEPES, 0.6 mM KH_2_PO_4_, 4.6 mM NaHCO_3_, 30 mM Taurine and 10 mM 2,3-butanedione monoxime (BDM)) for 4 min at 4 ml min^−1^ at 37 °C. The solution was then switched to digestion buffer (perfusion buffer containing 2.4 mg ml^−1^ Type II Collagenase, Worthington) for 3 min, followed by digestion buffer containing 40 μM CaCl_2_ for a further 8 min after which hearts were cut below the atria and placed in a sterile container filled with 5 ml digestion buffer. Following transfer to a laminar flow cell culture hood, ventricles were cut into small pieces and 10 ml myocyte stopping buffer (perfusion buffer containing 10% fetal bovine serum (FBS) and 12.5 μM CaCl_2_) added. The solution was pipetted up and down for 5 min using a plastic transfer pipette and placed in a 50-ml tube with an additional 5 ml stopping buffer, followed by centrifugation for 3 min at 20 *g* and resuspension of the pellet in 20 ml stopping buffer containing 2 mM ATP. Calcium was then reintroduced in three stages by repeated centrifugation (20 *g* for 3 min) and resuspension in 10 ml stopping buffer containing 100, 400 and 900 μM CaCl_2_. Following the final centrifugation, myocytes were resuspended in myocyte plating medium (Eagle's MEM w/ Hanks' balanced salt solution (HBSS) containing 10% FBS, 10 mM BDM, 100 U ml^−1^ penicillin-G, 2 mM Glutamine and 2 mM ATP) pre-equilibrated in a 37 °C 5% CO_2_ incubator and plated in 24-well plates on laminin-coated coverslips for 1 h. Plating medium was then removed and myocytes gently washed with pre-equilibrated myocyte culture medium (Eagle's MEM w/HBSS containing 1 mg ml^−1^ BSA, 10 mM BDM, 100 U ml^−1^ penicillin-G, 2 mM glutamine, 5 μg ml^−1^ insulin, 5 μg ml^−1^ transferring, 5 ng ml^−1^ selenium) and left for 24 h.

NRCMs were isolated from 1- to 3-day-old Sprague–Dawley rat neonates. Pups were killed by cervical dislocation followed by decapitation, then briefly rinsed in 70% ethanol for surface sterilization. Next, the hearts were collected into filter-sterilized ADS solution pH 7.35 (116 mM NaCl, 20 mM HEPES, 1 mM NaH_2_PO_4_, 5.5 mM glucose, 5.5 mM KCl and 1 mM MgSO_4_) on ice. Following transfer to a laminar flow cell culture hood, ventricles were dissected from extraneous tissue and atria, and cut into small pieces. Ventricular tissues were then digested through shaking at 37 °C in sterile ADS solution containing 0.6 mg ml^−1^ collagenase A (Roche) and 0.6 mg ml^−1^ pancreatin (Sigma) for 7 min. Cells were detached from the tissue by passing several times through a pipette, collected from the supernatant and discarded from this first digestion. The digestion process was then repeated a further seven times. Collected cells from digestions 2–8 were passed through a 70-μm cell strainer, to which 2 ml of FBS (Invitrogen) was added to neutralize the collagenase. The pooled cells were spun at 1,200 r.p.m. for 5 min and the pellet resuspended in 40 ml of pre-plating medium (68% DMEM, 17% M199, 10% horse serum, 5% FBS and 2.5 μg ml^−1^ amphotericin B). Cardiac fibroblasts were removed from the cell suspension through plating in 10 mm culture dishes for 1 h, to allow them to adhere, and then retrieving the myocytes in the media for counting. Cells were diluted to 1 × 10^6^ cells per ml with plating medium (as for pre-plating with the addition of 1 μM BRDU (5-bromo-2-deoxyuridine)) and incubated for 24 h at 37 °C following plating into 6-well BD Falcon Primaria tissue culture plates (for expression analysis, 2.5 × 10^6^ cells per well) or on laminin-coated coverslips in 24-well plates (for immunostaining, 5 × 10^5^ cells per well). The following day, cardiomyocytes were washed twice with PBS and kept in maintenance medium (80% DMEM and 20% Medium 199, 1% FBS, 2.5 μg ml^−1^ amphotericin B and 1 μM BRDU) at 37 °C.

### Isolation of ACFs

ACFs were isolated from 3- to 4-month-old mice. Mice were killed by cervical dislocation and their hearts were rapidly removed. The hearts were then mashed with scalpels and digested with 10 ml collagenase solution (120 mg collagenase A (Roche) and 12 mg protease (Sigma) dissolved in 80 ml PBS solution) at 37 °C for 5 min, for three times. Cells were collected after each digestion and the collagenase was deactivated by addition of FBS solution. The harvested fibroblasts were centrifuged for 5 min at 220 *g* (Beckman Coulter Allegra 6R). The cell pellet was resuspended in 10 ml ACF media (80% DMEM, 20% FBS, 1% penicillin/streptomycin, 1% Fungizone and 1% non-essential amino acids). Fibroblasts were then plated in 10 ml BD Primaria tissue culture plates overnight. The next day, the media was removed and replaced with 10 ml ACF media.

### Cellular hypertrophy experiments

NRCM were cultured in cardiomyocyte maintenance medium (68% DMEM, 17% M199, 10% horse serum, 5% FBS, 2.5 μg ml^−1^ amphotericin B and 1 μM BrdU), whereas adult rat cardiomyocytes were cultured in pre-equilibrated myocyte culture medium (Eagle's MEM w/HBSS containing; 1 mg ml^−1^ BSA, 10 mM BDM, 100 U ml^−1^ penicillin-G, 2 mM Glutamine, 5 μg ml^−1^ insulin, 5 μg ml^−1^ transferring and 5 ng ml^−1^ selenium) for 24 h. Next, the medium was replaced with conditioned medium from ACFs culture. To prepare the conditioned medium, ACFs isolated from WT or *PMCA4*^*−/−*^ mice were plated in cardiomyocyte maintenance medium for 24 h. The media were collected and used to culture NRCM. Cells were then stimulated with phenylephrine (30 μM) for 72 h with or without 0.2 μg ml^−1^ anti-sFRP2 antibody (Abcam) for 72 h. To specifically visualize cardiomyocytes, cells were stained with anti-α-actinin antibody (Sigma). The cell size was then measured using ImageJ software (NIH). The level of sFRP2 in the conditioned medium of WT or *PMCA4*^*−/−*^ ACFs was determined using ELISA (My BioSource) following the m manufacturer's protocol.

### Intracellular calcium measurement

Intracellular calcium measurement was carried out as described before^24^. Briefly, 10^4^ ACFs were plated in BD Primaria 96-well plates and incubated at 37 °C for 24 h. The medium was replaced by 80 μl of loading solution (5 μM Fluo-3 acetoxylmethyl+0.1% Pluronic F.127 in HBSS solution) and incubated at 37 °C for 30 min. Next, the medium was replaced by 100 μl of fresh HBSS–BSA–probenecid solution (HBSS, 1% w/v BSA and 2.5 mM probenecid) and were incubated for 30 min at 37 °C. Cells were then washed with 100 μl of HBSS–BSA–probenecid and finally maintained in 80 μl of fresh HBSS–BSA–probenecid containing 1.26 mM calcium chloride. Baseline fluorescence (F) was measured for 3 min with filters for excitation at 485 nm and for emission at 538 nm using a BMG FLUOstar Omega plate reader. Following baseline fluorescence measurement, 14 μl of *F*_min_ solution (HBSS–BSA–probenecid pH 7.45, 100 μM ionomycin, 10 μM Thapsigargin and 20 mM EGTA pH 8.0) was added to each well, to deplete the calcium from the cells and leave only the autofluorescence, which was measured for 120 min (*F*_min_). Finally, 16 μl of *F*_max_ (250 mM calcium chloride in HBSS–BSA–probenecid) solution was added to saturate the cells with calcium and fluorescence was measured for another 5 min (*F*_max_).

### Western blotting and immunofluorescence

Protein was extracted from pelleted suspended or adherent cells through lysis in RIPA buffer (PBS containing 1% IGEPAL CA-630, 0.5% sodium deoxycholate, 0.1% SDS, 0.5 mM phenylmethylsulphonyl fluoride, 500 ng ml^−1^ Leupeptin, 1 μg ml^−1^ Aprotinin and 2.5 μg ml^−1^ Pepstatin A). Harvested tissue stored at −80 °C since collection was cut into small pieces and homogenized in RIPA buffer in a dounce homogenizer. Cellular debris was removed from protein extracts by centrifugation (3,000 r.p.m. for 5 min at 4 °C) and the supernatant stored at −80 °C. Protein concentration was determined using a bicinchoninic acid assay kit (Pierce) as per the manufacturer's instructions.

Western blot analysis was conducted using a standard protocol. Proteins were separated by SDS–PAGE using 8% gels, transferred to Immobilon-polyvinylidene difluoride membrane (Millipore) and blocked in 4% BSA or 5% non-fat milk. Primary antibodies used were: anti-PMCA4 (clone JA9) (Abcam; 1:1,000 for western blotting, 1:100 for immunofluoresence), anti-DDR2 (Abcam; 1:100), anti-sFRP2 (Abcam; 1:1,000), anti-active β-Catenin (Millipore; 1:500), anti-GAPDH (Abcam; 1:5,000) anti-β-actin (Abcam; 1:5,000) and anti-α-tubulin (Abcam; 1:5,000). We used horseradish peroxidase-labelled secondary antibodies (Cell Signaling; 1:5,000) and detected signal using enhanced chemiluminescence (GE Healthcare) in a ChemiDoc XRS+ Imaging System (Biorad). Full blots of cropped images are provided in Supplementary Fig. 14.

For immunofluorescence, anti-α-actinin (Sigma; 1:100) and anti-phalloidin (Molecular Probes; 1:100) antibody were used. We used Texas red or fluorescein isothiocyanate-labelled secondary antibodies (Jackson ImmunoResearch) for visual detection.

### Histology

Heart tissues were fixed in 4% paraformaldehyde in PBS, embedded in paraffin and sectioned at 5 μm thickness. Haematoxylin and eosin staining and Masson's trichrome stains were performed using standard procedures. Cross-sectional cardiomyocyte size and percentage of interstitial fibrosis were assessed in haematoxylin and eosin-, and Masson's trichrome-stained sections, respectively, using ImageJ software.

### RNA isolation and real-time RT–PCR

Total RNA was prepared from freshly isolated heart tissues or cultured ACFs using Trizol reagent (Invitrogen) following the manufacturer's instructions. We used the QuantiTect-SYBR Green RT–PCR system (Qiagen) for real-time qRT–PCR analysis. Data were calculated using the ΔΔCt method and are presented as fold induction of target gene transcripts relative to control group. Reactions were performed in an ABI 7500 Fast System (Applied Biosystems). The following primers were purchased from Qiagen (Quantitect Primer Assay system): ANP, Mm_LOC230899_1_SG; BNP, Mm_Nppb_1_SG; SFRP2 Mm_Sfrp2_1_SG; TCF4, Mm_Tcf4_va.1_SG; Pax2, Mm_Pax2_1_SG; and GAPDH, Mm_Gapdh_2_SG. Threshold cycle (Ct) values were determined by using the Sequence Detection System software. GAPDH levels were used as a reference.

### Microarray analysis

RNA was obtained and pooled from three batches of fibroblasts isolated either from *PMCA4*^*−/−*^ or WT mice. We used three independent RNA pools of each genotype isolated with Trizol (Invitrogen) following the manufacturer's instructions. All the following steps were conducted by the genomics and bioinformatics core facilities at the University of Manchester following their standard protocols. Briefly, we first assessed the purity of RNA using an Agilent Bioanalyzer 2100 (Agilent Technologies). Cochlear complementary RNA (cRNA) was prepared by sequentially generating complementary DNA with the one-Cycle cDNA Synthesis Kit and used for hybridization to MOE430A Genechips (Affymetrix) and then cRNA was purified and used as a template for the *in vitro* transcription reaction for cRNA amplification and biotin labelling. This was followed by hybridization to the GeneChip arrays and scanned with a GeneChip Scanner 3000 7G–4C (Affymetrix). The analysis was initially performed with MAS5.0 (Affymetrix) and Robust Multiarray Average software^45^, which indicated high variability that was associated with the biological variability and nonspecific hybridization. Further analysis was then conducted using the software package PUMA (Propagating Uncertainty in Microarray Analysis), to estimate the gene expression levels. To reduce the number of false positives, the analysis of the fold change was used in combination with the Probability of Positive Log Ratio (PPLR) algorithm from the PUMA package^46^.

### Luciferase assay

The luciferase reporter for assessing β-catenin activity, which contains TCF/LEF sites was a gift from Dr Randall Moon (Addgene plasmid #12456)^19^. The reporter cassette containing NF-κB-binding sites was obtained from Clontech. Constructs were cloned into the pAd-DEST Gateway vector (Invitrogen), to produce adenoviruses expressing these reporter genes.

For luciferase assay, 7 × 10^5^ NRCMs were plated in 24-well plates in 1 ml maintenance media for 24 h. Next, the media was changed with either maintenance media or fibroblasts conditioned media. Adenovirus-encoding the TCF/LEF-luciferase construct was added and cells were then stimulated with 30 μM phenylephrine for 72 h. Luciferase assays were performed using luciferase assay reagent (Promega) following manufacturer's recommended protocol.

The NF-κB-luciferase reporter was used to examine NF-κB activity in cardiac fibroblasts. In brief, 5 × 10^4^ ACFs were plated in 24-well plates for 24 h. Next, the cells were infected with the NF-κB reporter adenovirus and incubated for 72 h at 37 °C. Luciferase assays were performed using luciferase assay reagent (Promega) following the manufacturer's recommended protocol.

### Whole-body phenotypic analysis

The study was performed on 12 male mice: 6 KOs and 6 control WTs. Phenotyping began when mice were 15 weeks of age. Food consumption and body weight were monitored once a week between 16 and 21 weeks of age. Dysmorphology screen including body observation, physical appearance and general behaviour was performed at 17 weeks of age. Blood was collected by retro-orbital puncture after overnight fasting under isoflurane anaesthesia at the age of 19 weeks for basic chemistry, blood lipid and haematology analysis. At 20 weeks of age, indirect calorimetry was performed to evaluate energy expenditure. At 21 weeks of age, blood was collected by retro-orbital puncture for immunology (plasma IgG, IgA and IgM measurement) and enzymatic activities analysis. Bone mineral density, body lean and fat content were evaluated by Dexascan analysis at the age of 22 weeks. X-ray analysis was performed at the same age. At 24 and 26 weeks of age, blood was collected by retro-orbital puncture for metabolic and endocrine exploration. At the end of the study (30-week-old mice), blood was collected by intra-cardiac puncture for coagulation tests.

### Statistical analysis

Data are expressed as mean+s.e.m. Student's *t*-test or one-way analysis of variance followed by *post-hoc* multiple comparison were used where appropriate. The probability level for statistical significance was set at *P*<0.05.

## Additional information

**Accession codes:** Microarray data have been deposited in NCBI's Gene Expression Omnibus under accession code GSE77359 (http://www.ncbi.nlm.nih.gov/geo/query/acc.cgi?acc=GSE77359).

**How to cite this article:** Mohamed, T. M. A. *et al*. The plasma membrane calcium ATPase 4 signalling in cardiac fibroblasts mediates cardiomyocyte hypertrophy. *Nat. Commun.* 7:11074 doi: 10.1038/ncomms11074 (2016).

## Supplementary Material

Supplementary Information Supplementary Figures 1-14 and Supplementary Tables 1-4
