# Supplementary material for: The plasma membrane calcium ATPase 4 signalling in cardiac fibroblasts mediates cardiomyocyte hypertrophy
Source: Nat Commun. 2016 Mar 29;7:11074. doi: 10.1038/ncomms11074 (PMC4820544; doi:10.1038/ncomms11074)
Supplement: Supplementary Information — Supplementary Figures 1-14 and Supplementary Tables 1-4 [file ncomms11074-s1.pdf]

Supplementary Information

Supplementary Figure 1

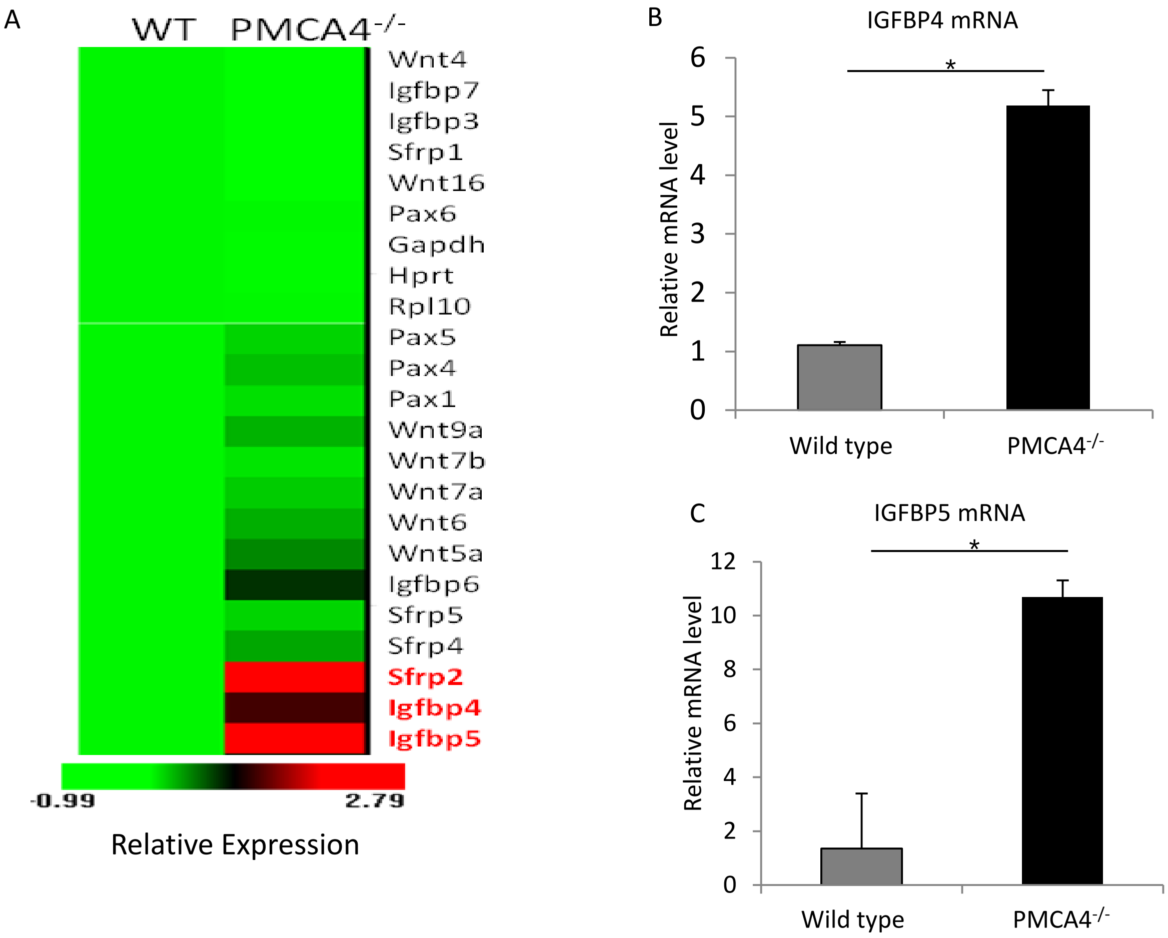

Supplementary figure 1 A) Microarray heat map comparing mRNA expression of several genes involved in regulating the Wnt pathway between WT and *PMCA4*$^{-/-}$ fibroblasts. B) qRT-PCR analysis showing increased IGFBP4 and (C) IGFBP5 mRNA levels in *PMCA4*$^{-/-}$ cardiac fibroblasts (n=4 in each group, *P<0.05, Student's t-test). All error bars represent the SEM.

Supplementary Figure 2

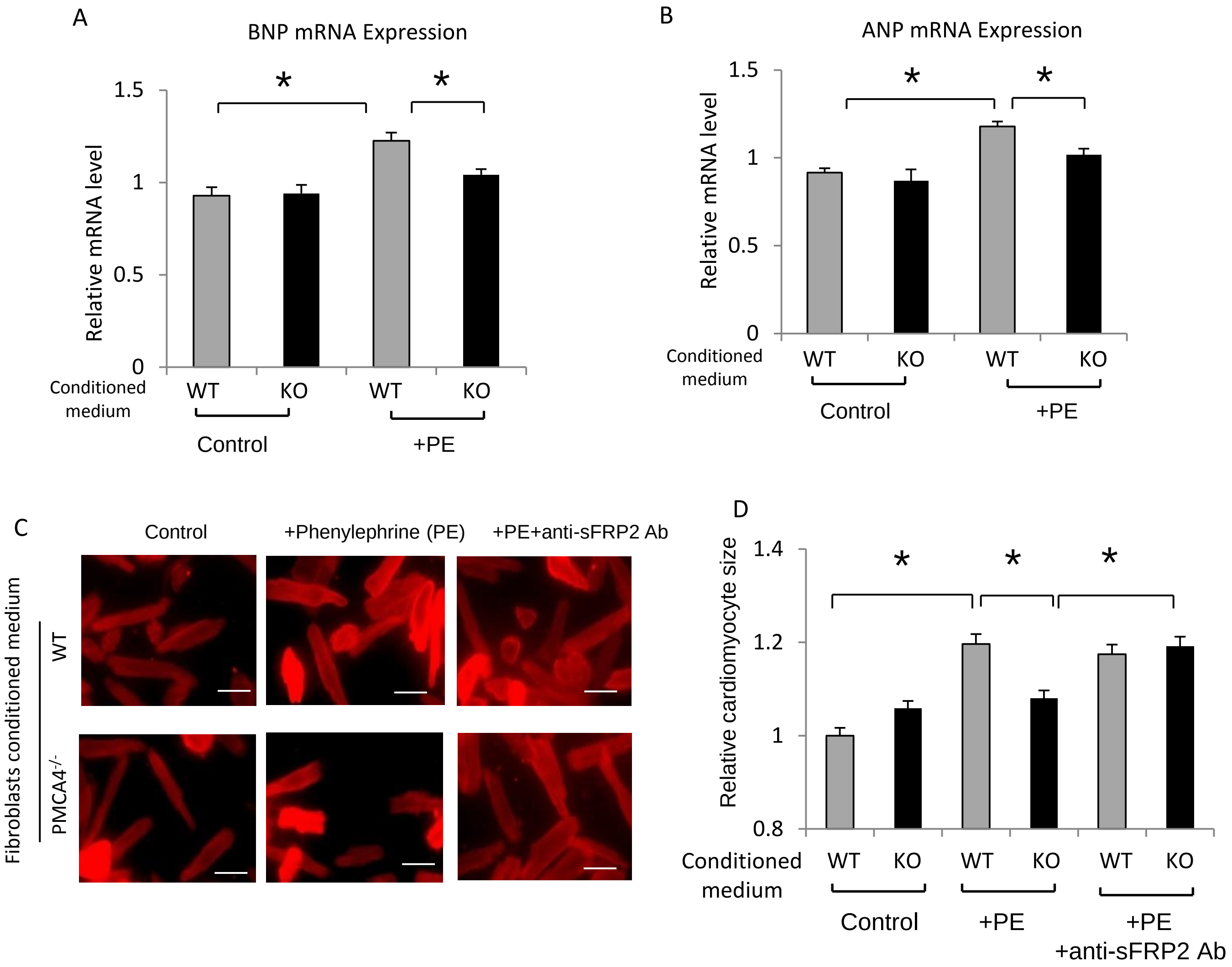

Supplementary figure 2 A) Analysis of BNP and (**B**) ANP expression in isolated neonatal rat cardiomyocytes (NRCM) cultured in conditioned medium of either WT or *PMCA4*$^{-/-}$ cardiac fibroblasts. NRCM were treated with phenylephrine (PE), 30 µM for 72 hours (*P<0.05, One way Anova followed by posthoc multiple comparison). **C**) Representative images of adult rat cardiomyocytes (ARCM) cultured in conditioned medium of either WT or *PMCA4*$^{-/-}$ cardiac fibroblasts and then treated with phenylephrine (PE), 30 µM or PE with anti-sFRP2 antibody (0.2 µg per ml) for 48 hours (Scale bar = 50µm). **D**) Analysis of cell surface area showed that treatment with *PMCA4*$^{-/-}$ fibroblasts conditioned medium significantly reduced PE-induced hypertrophy and addition of anti-sFRP2 antibody abolished the anti-hypertrophic effect of *PMCA4*$^{-/-}$ conditioned medium (Results were from 3 independent experiments; *P<0.05, One way Anova followed by posthoc multiple comparison). All error bars represent the SEM.

Supplementary Figure 3

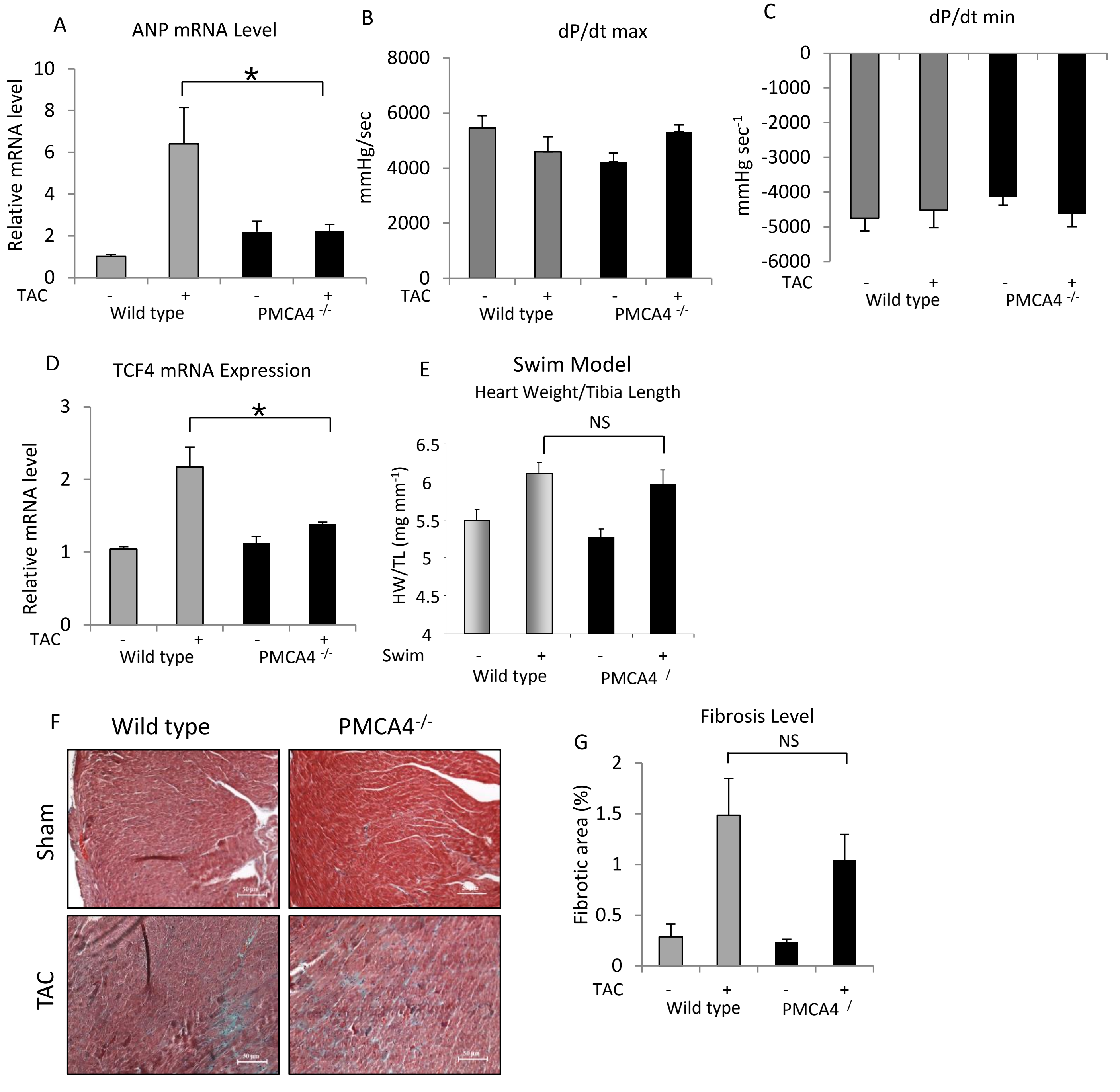

Supplementary figure 3 A) Expression of hypertrophic marker ANP in the hearts of WT and *PMCA4*$^{-/-}$ mice following TAC (WT Sham, n=4, WT TAC, n=4, KO sham n=5, KO TAC, n=4; *P<0.05, one way Anova). **B**) Analysis of dP/dt_{max} and (**C**) dP/dt_{min} showed no difference in cardiac contractility between WT and *PMCA4*$^{-/-}$ mice after 5 weeks of TAC (n= 10 in each group). **D**) Expression of TCF4, a β-catenin target gene, was significantly reduced in *PMCA4*$^{-/-}$ TAC mice compared to WT-TAC group (WT Sham, n=4, WT TAC, n=4, KO sham n=5, KO TAC, n=4;*P<0.05, one way Anova). **E**) Analysis of heart weight/tibia length ratio following 4 weeks swimming exercise showed no difference between WT and *PMCA4*$^{-/-}$ mice (n=7 in each group). **F)** Representative images of Masson's trichrome staining and **(G)** quantification of fibrotic area indicated that there was no difference in the fibrosis level between *PMCA4*$^{-/-}$ and WT controls (scale bars= 50μm; NS=not significant). Error bars represent SEM.

Supplementary Figure 4

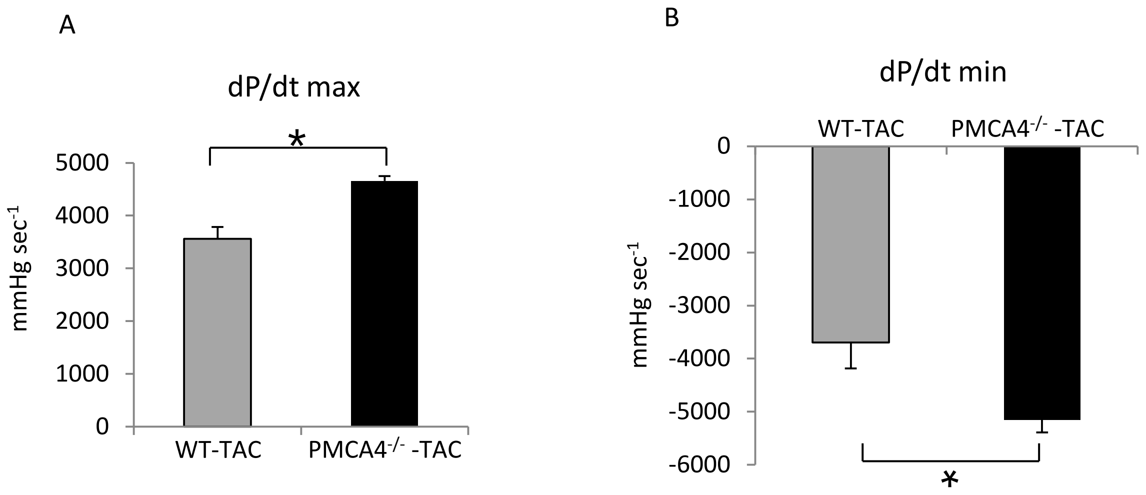

Supplementary figure 4 Hemodynamic analysis to assess **A**) dP/dt $_{max}$ and **B**) dP/dt $_{min}$ after 12 weeks of TAC suggested that *PMCA4*$^{-/-}$ mice exhibited better contractile function compared to WT after 12 weeks of TAC (WT TAC, n=3, *PMCA4*$^{-/-}$ TAC, n=-5; *$P<0.05$, Student's t-test). All error bars represent the SEM.

Supplementary Figure 5

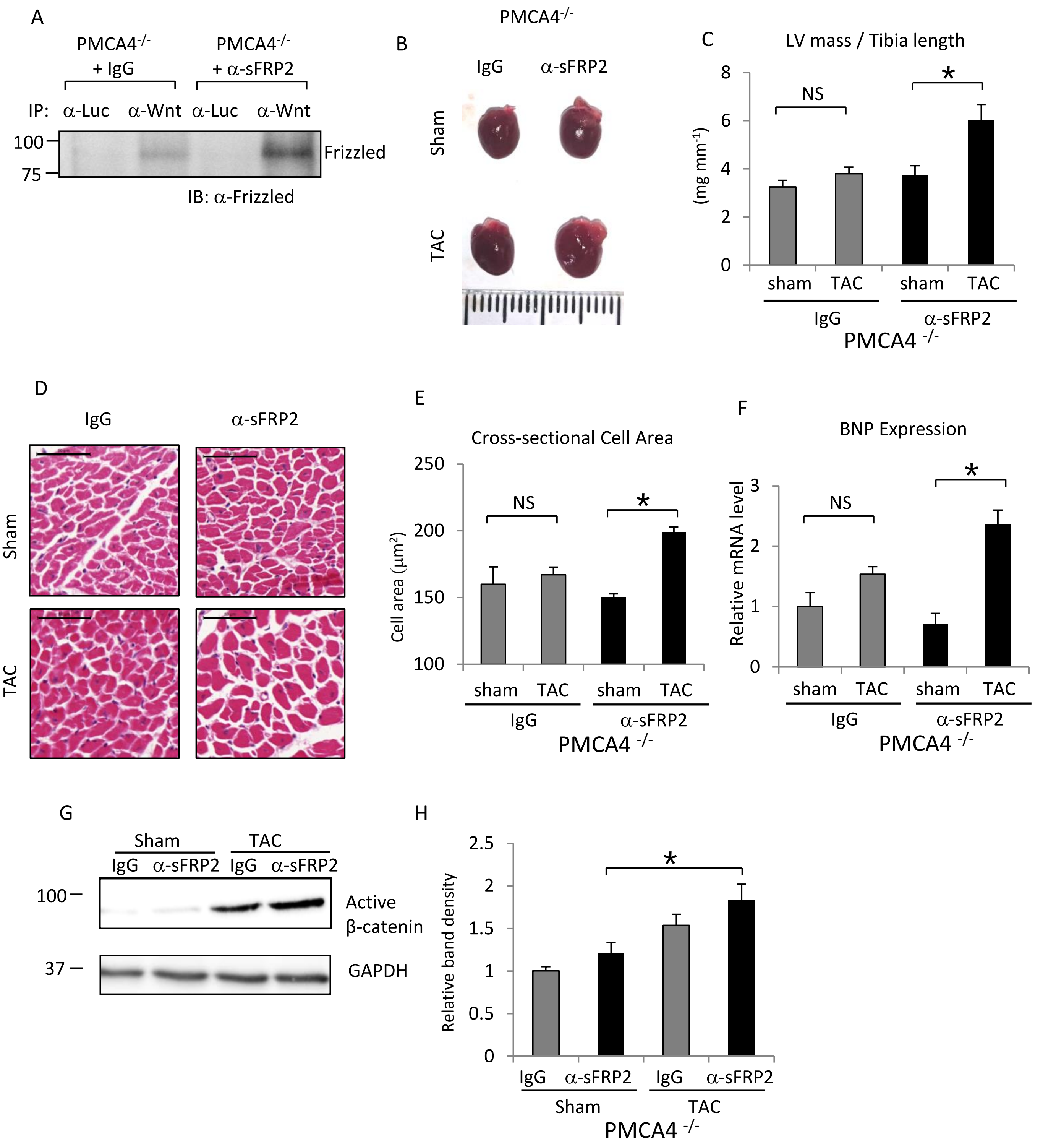

Supplementary Figure 5 Treatment with anti-sFRP2 antibody restored the hypertrophic response to TAC in *PMCA4$^{-/-}$* mice A) Immunoprecipitation analysis suggested that PMCA4$^{-/-}$ mice treated with anti-sFRP2 antibody (200 μg per kg BW) showed an increase level of Wnt-Frizzled binding in the heart compared with mice treated with control IgG. 100 μg of total heart extracts were immunoprecipitated with 1 μg of indicated antibodies in the presence of Protein A/G agarose. Western blot was conducted to detect Frizzled in the precipitated proteins **B)** Images of hearts from *PMCA4$^{-/-}$* mice following sham or TAC surgery and treatment with goat anti-sFRP2 antibody (α-sFRP2) or normal goat IgG (200 μg per kg BW per day) intraperitoneally. **C)** Quantification of left ventricular mass normalised to tibia length suggested a significant increase in hypertrophic response to TAC in *PMCA4$^{-/-}$* mice treated with α-sFRP2 (IgG+sham, n=6; IgG+TAC, n=3; α-sFRP2+sham, n=6, α-sFRP2+TAC, n=5; *P<0.05, NS=not significant). **D)** Analysis of hematoxylin-eosin stained histological sections and **(E)** measurement of cardiomyocyte cross sectional area showed that treatment with α-sFRP2 restored the hypertrophic response in *PMCA4$^{-/-}$* mice (scale bar = 50 µm,*P<0.05). **F)** Analysis of BNP mRNA level by qRT-PCR, **(G)** western blot analysis of the level of β-catenin activation as well as **(H)** quantification of band density of active β-catenin supported the finding that α-sFRP2 treatment increased the hypertrophic response in *PMCA4$^{-/-}$* mice (*P<0.05). One way Anova followed by posthoc multiple comparison used for all statistics, error bars represent the SEM.

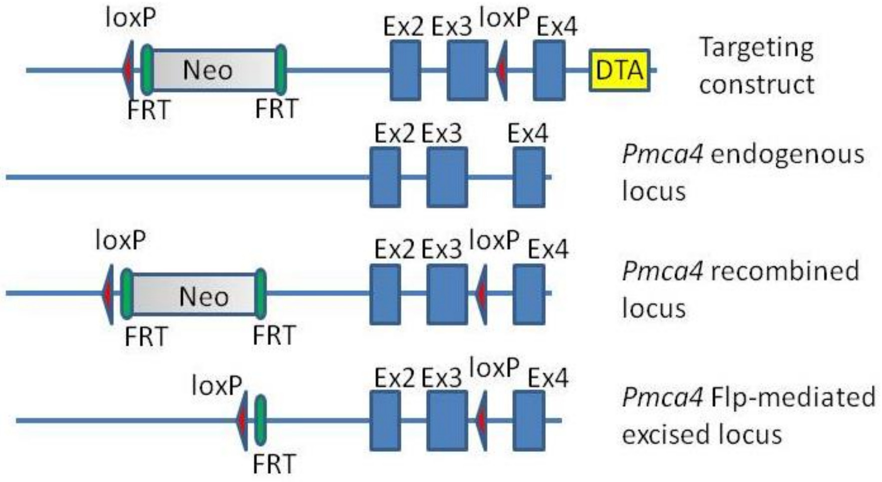

Supplementary figure 6 Schematic diagram showing the strategy to produce *PMCA4*$^{flox/flox}$ animals.

Supplementary Figure 7

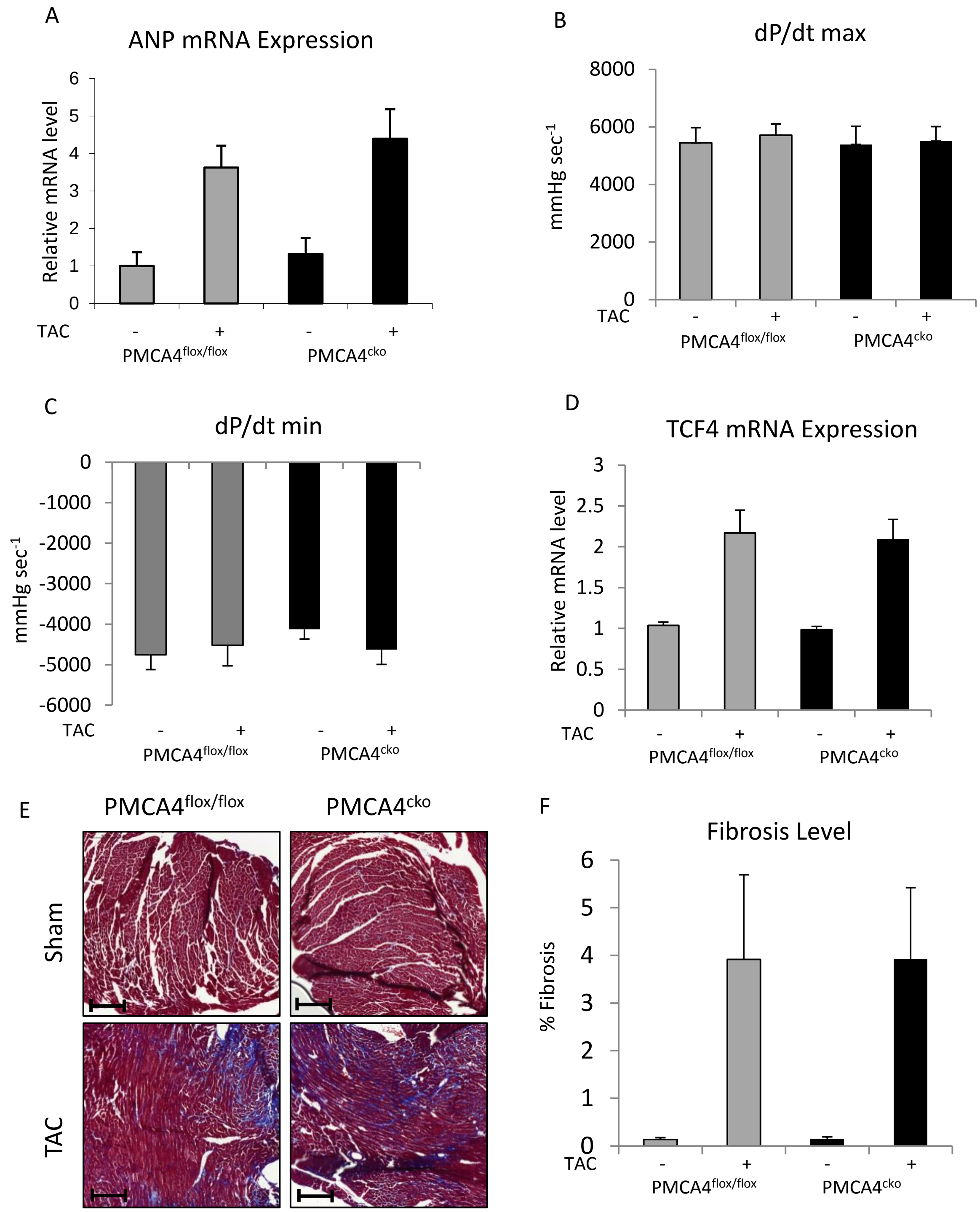

Supplementary figure 7 A) mRNA expression of ANP was not significantly different between *PMCA4*^{cko} and control mice (WT Sham, n=3, WT TAC, n=4, *PMCA4*^{cko} sham, n=5, *PMCA4*^{cko} TAC, n=5). **B**) dP/dt$_{max}$ and (**C**) dP/dt$_{min}$ values were not different between *PMCA4*^{cko} and controls (control sham, n=7; control TAC, n=9; *PMCA4*^{cko} sham, n=6; *PMCA4*^{cko} TAC, n=7). **D**) The level of TCF4 expression detected by qRT-PCR showed no difference between *PMCA4*^{cko} and controls (WT Sham, n=3, WT TAC, n=4, *PMCA4*^{cko} sham, n=5, *PMCA4*^{cko} TAC, n=5). **E)** Images of Masson's trichrome staining (scale bar = 200μm) and **(F)** analysis of fibrosis level showed that there was no difference between *PMCA4*^{cko} and control mice. All error bars represent the SEM.

Supplementary Figure 8

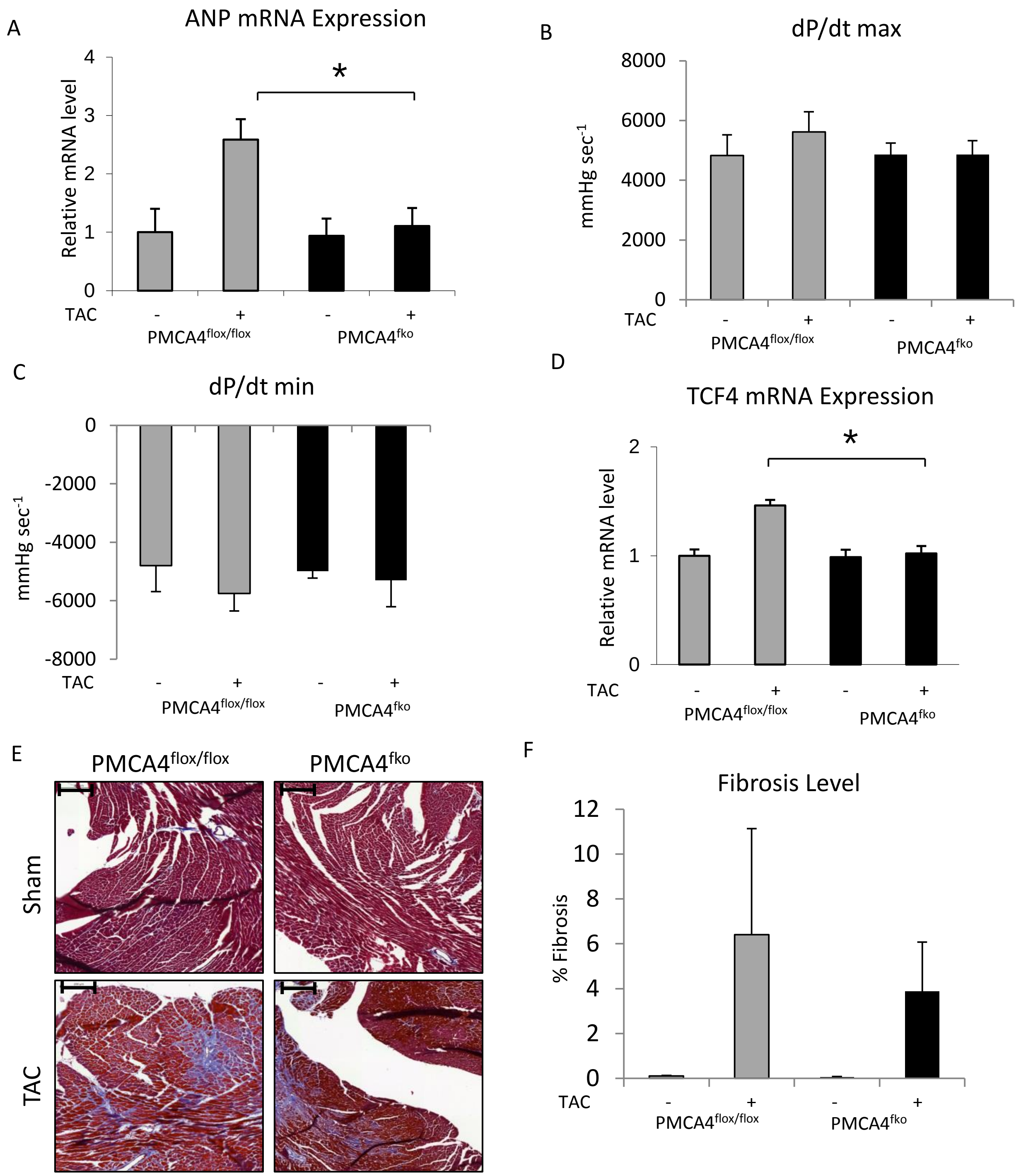

Supplementary figure 8 A) Analysis of ANP expression using qRT-PCR showed that cardiac expression of ANP was significantly lower in *PMCA4*^{fko} mice vs controls after TAC (control sham, n=4; control TAC, n=5; *PMCA4*^{fko} sham, n=4; *PMCA4*^{fko} TAC, n=4, *P<0.05, One way Anova). **B**) Analysis of dP/dt_{max} and (**C**) dP/dt_{min} did not show any difference of contractility between *PMCA4*^{fko} and control group . **D**) TCF4 expression was significantly reduced in *PMCA4*^{fko} mice vs controls (control sham, n=4; control TAC, n=5; *PMCA4*^{fko} sham, n=4; *PMCA4*^{fko} TAC, n=4, *P<0.05, One way Anova). **E)** Masson's trichrome staining (scale bar = 200μm) and **(F)** analysis of fibrosis level showed that there was no significant difference in fibrosis level between *PMCA4*^{fko} and control mice. All error bars represent the SEM.

Supplementary Figure 9

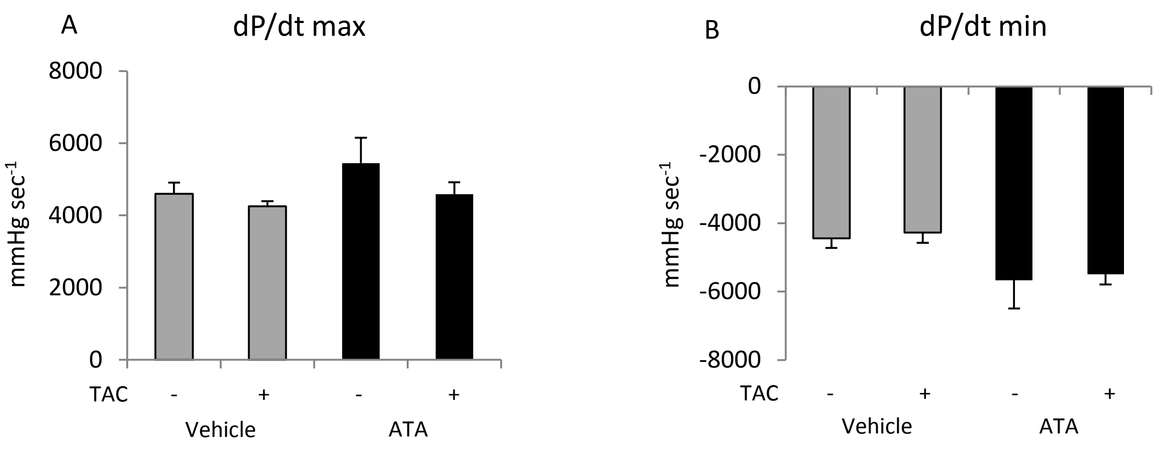

Supplementary figure 9 Cardiac function as indicated by **A**) dP/dt_{max} and **B**) dP/dt_{min} values did not differ between mice treated with vehicle and mice treated with ATA after 2 weeks of TAC (Sham + vehicle, n=10, TAC + vehicle, n=10, sham + ATA, n=5, TAC + ATA, n=6). All error bars represent the SEM.

Supplementary Figure 10

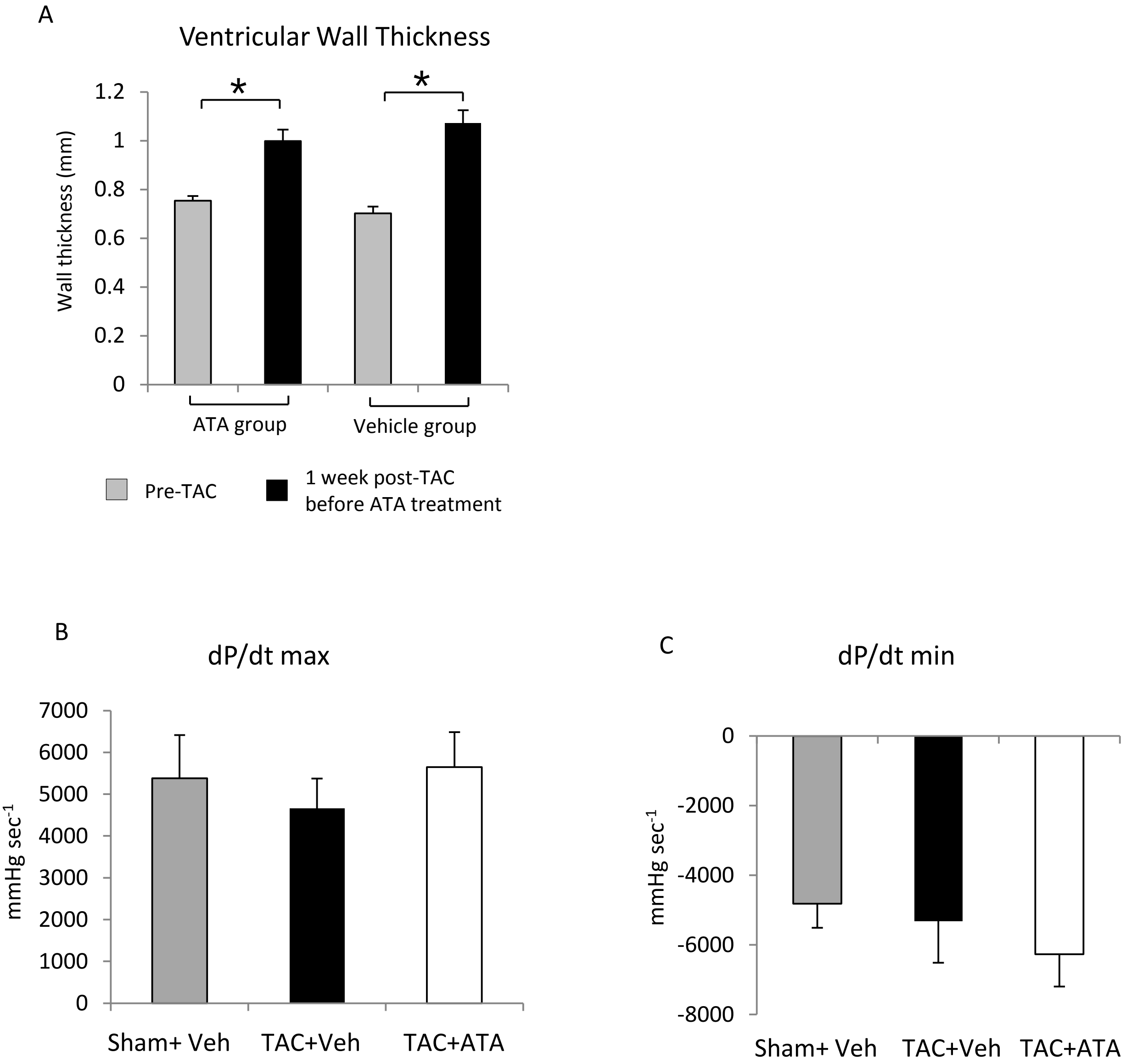

Supplementary figure 10 A) Echocardiography analysis to assess ventricular wall thickness (average of posterior and septal wall thickness) indicated a significant level of cardiac enlargement 1 week after TAC and before ATA treatment in both ATA group and vehicle group (ATA, n=6, vehicle, n=4, *P<0.05, paired t-test). **B**) Hemodynamic analysis revealed that both dP/dt max and (**C**) dP/dt min values did not differ between ATA-treated and vehicle-treated mice in the reversal treatment strategy (Sham+vehicle, n=4, TAC + vehicle, n=4, TAC + ATA, n=6). All error bars represent the SEM.

Supplementary Figure 11

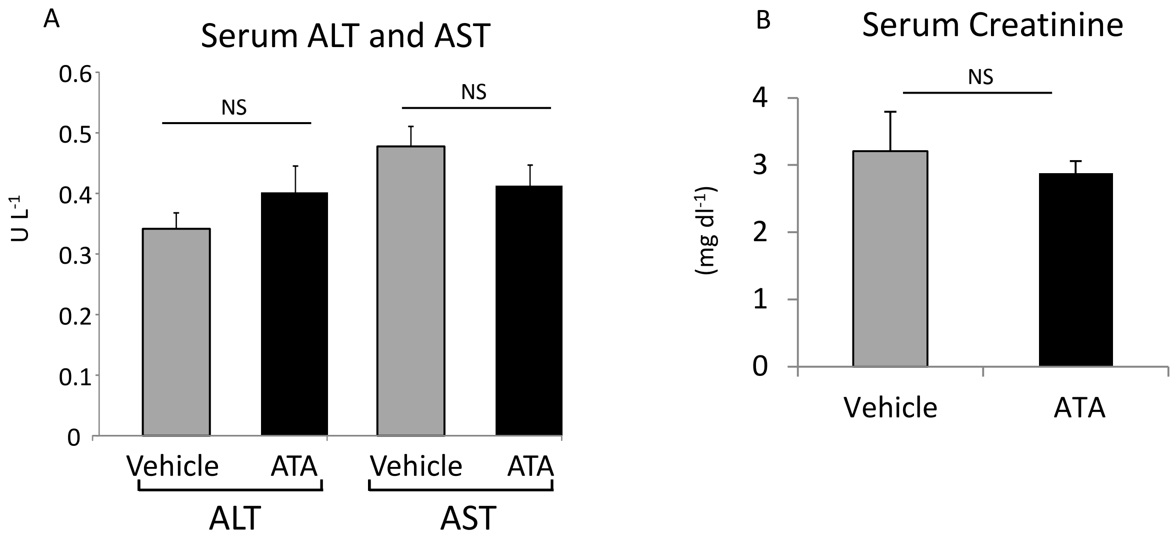

Supplementary figure 11 Analysis of **(A)** alanine transaminase (ALT) and aspartate transaminase (AST) and **B)** creatinine level in the serum of C57Bl/6 mice treated with vehicle or ATA (5mg per kg BW per day) for 4 weeks. ALT, AST and creatinine levels were detected using commercially available kit (Abcam). (Vehicle, n=4, ATA, n=5; NS=not statistically significant by student's t-test). All error bars represent the SEM.

Supplementary Figure 12

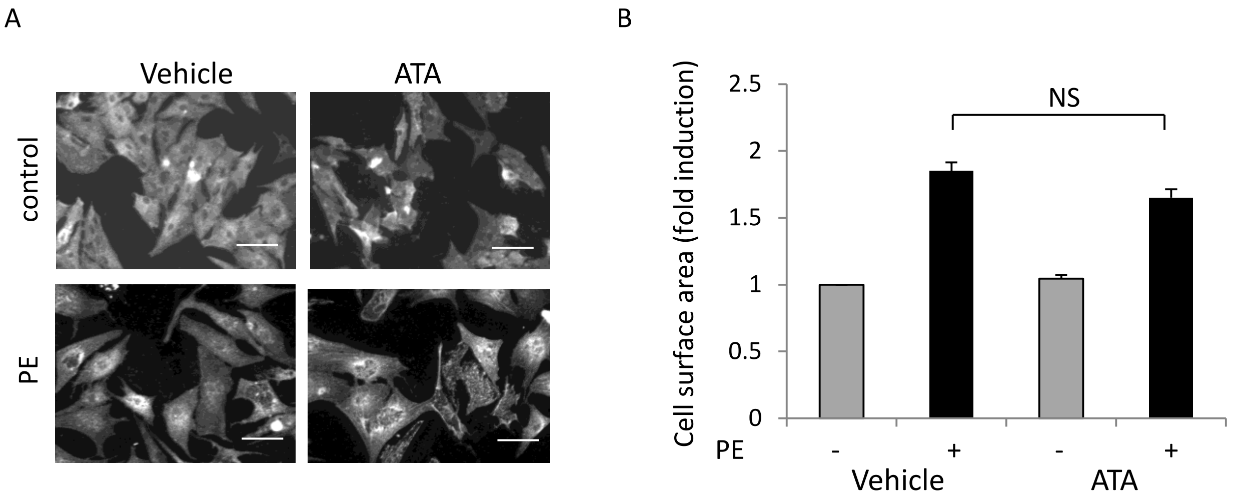

Supplementary figure 12 A) Images of neonatal rat cardiomyocytes treated with vehicle or ATA (1 μM) in the presence or absence of phenylephrine (30 μM) for 72 hours. **B)** Analysis of cell surface area indicated that there was no significant difference in hypertrophic response following ATA treatment (n=3 independent experiments). Scale bar = 25μm, NS = not significant (One way Anova). All error bars represent the SEM.

Supplementary Figure 13

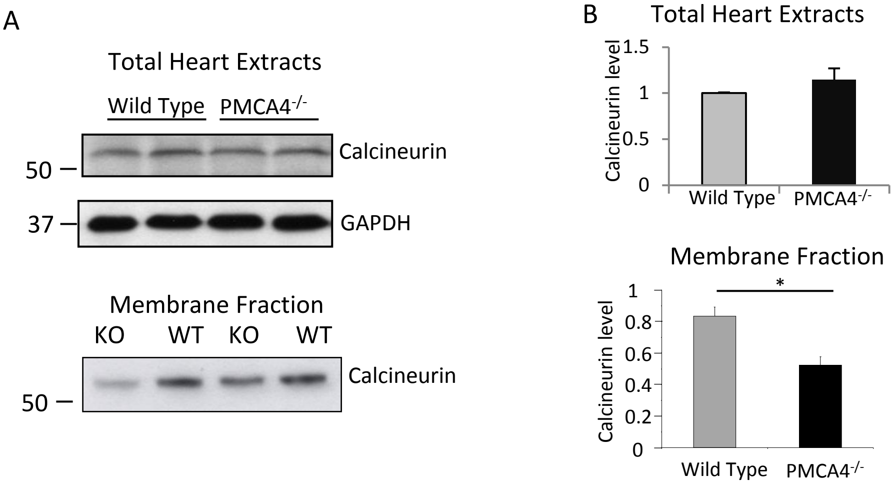

Supplementary figure 13 A) Western blot analysis of calcineurin level in total heart extracts and in the membrane fraction of *PMCA4*$^{-/-}$ mice compared to WT littermates. (**B**) Quantification of band density indicated that calcineurin level at the membrane compartment was significantly reduced in *PMCA4*$^{-/-}$ hearts, however total calcineurin level was not different (n= 6,*P<0.05, student’s t-test). All error bars represent the SEM.

Figure 1C

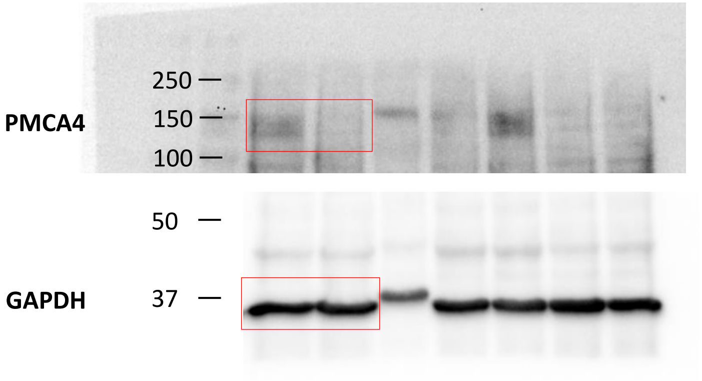

Figure 1F

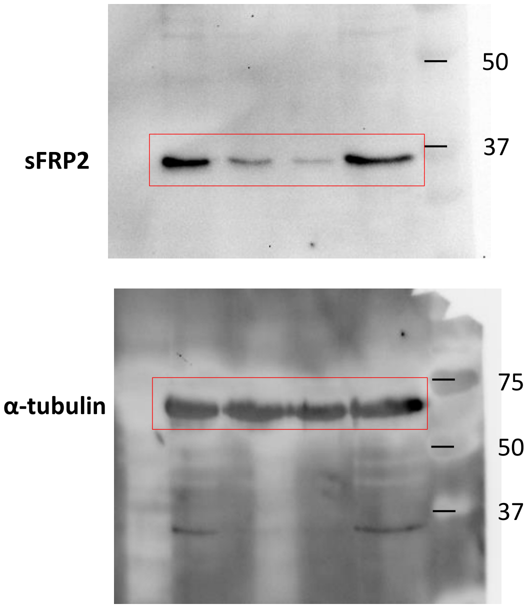

Figure 3F

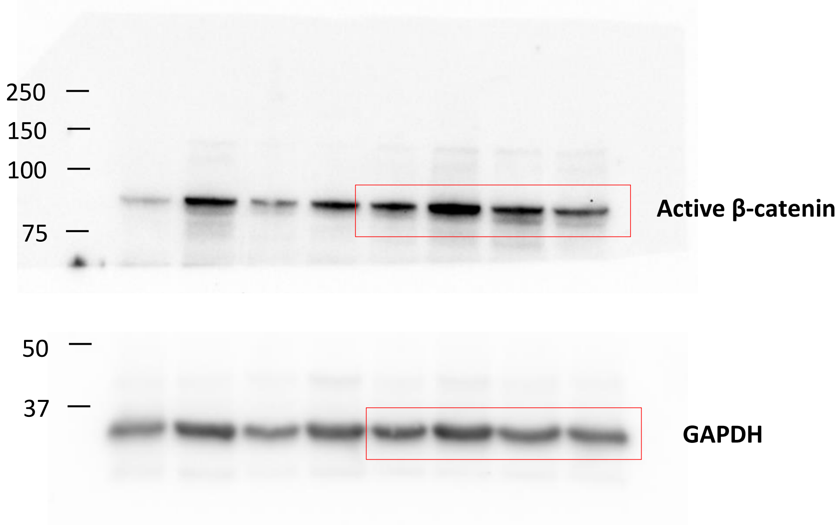

Figure 4B

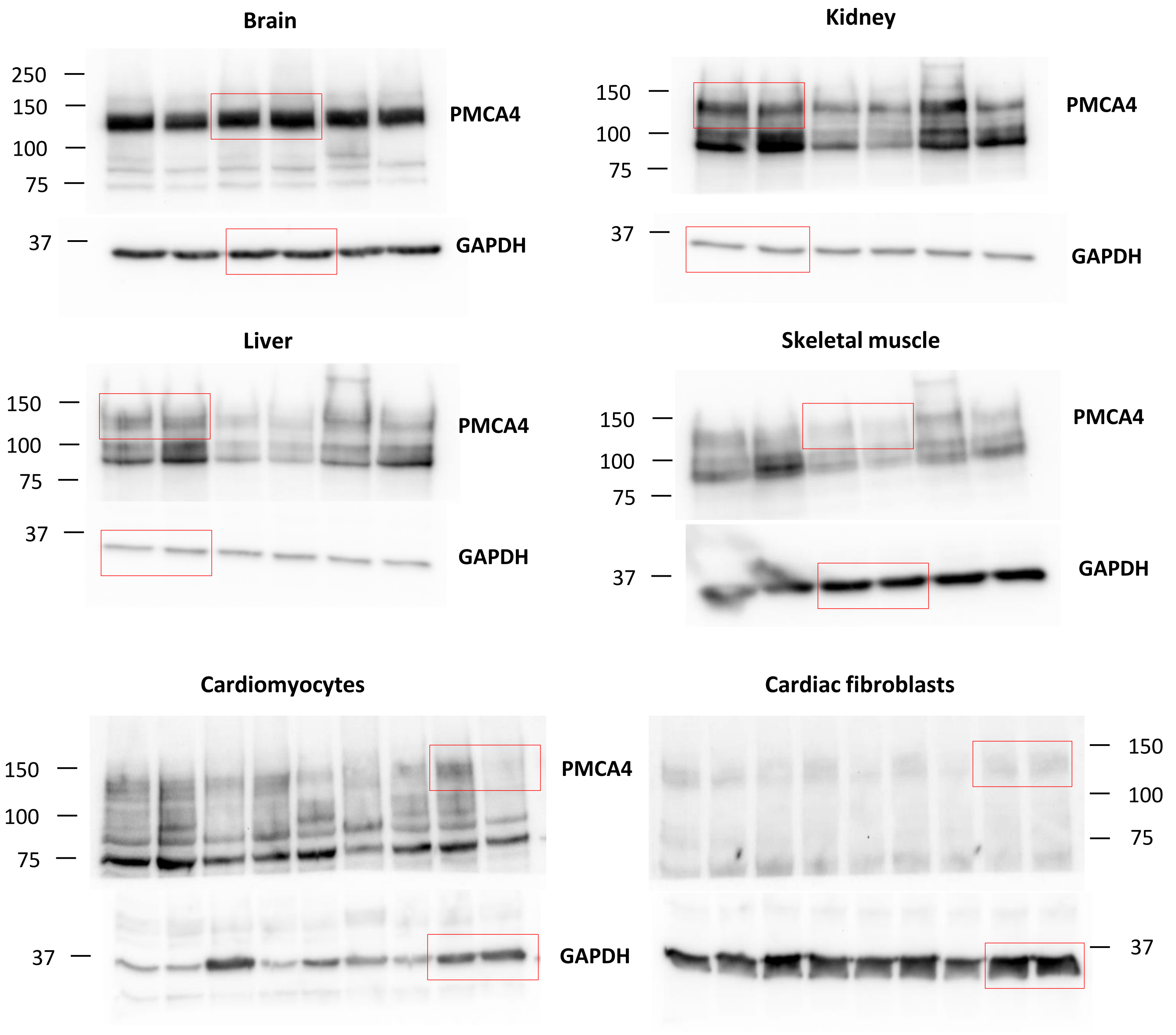

Figure 4J

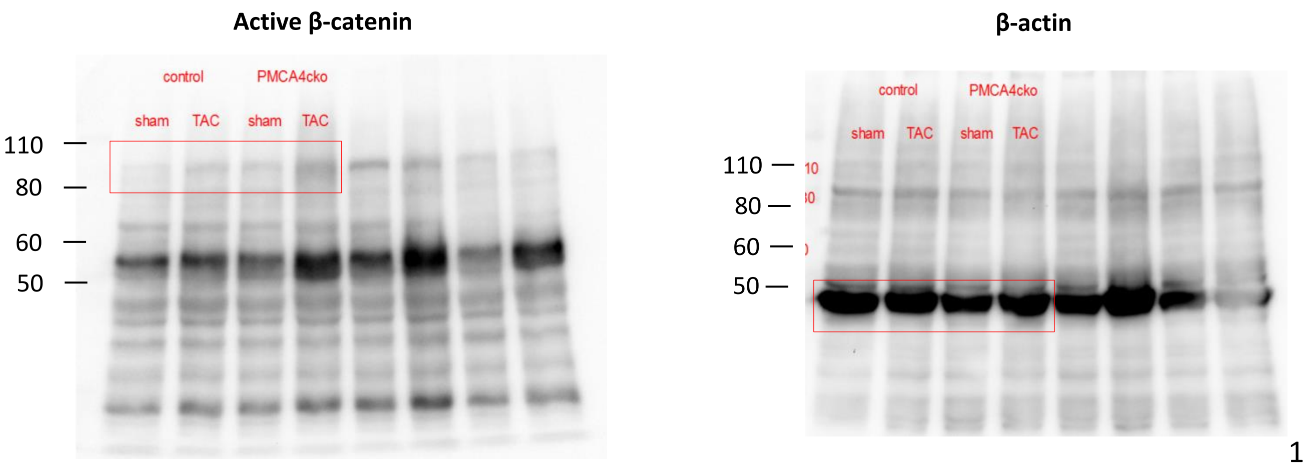

Figure 5B

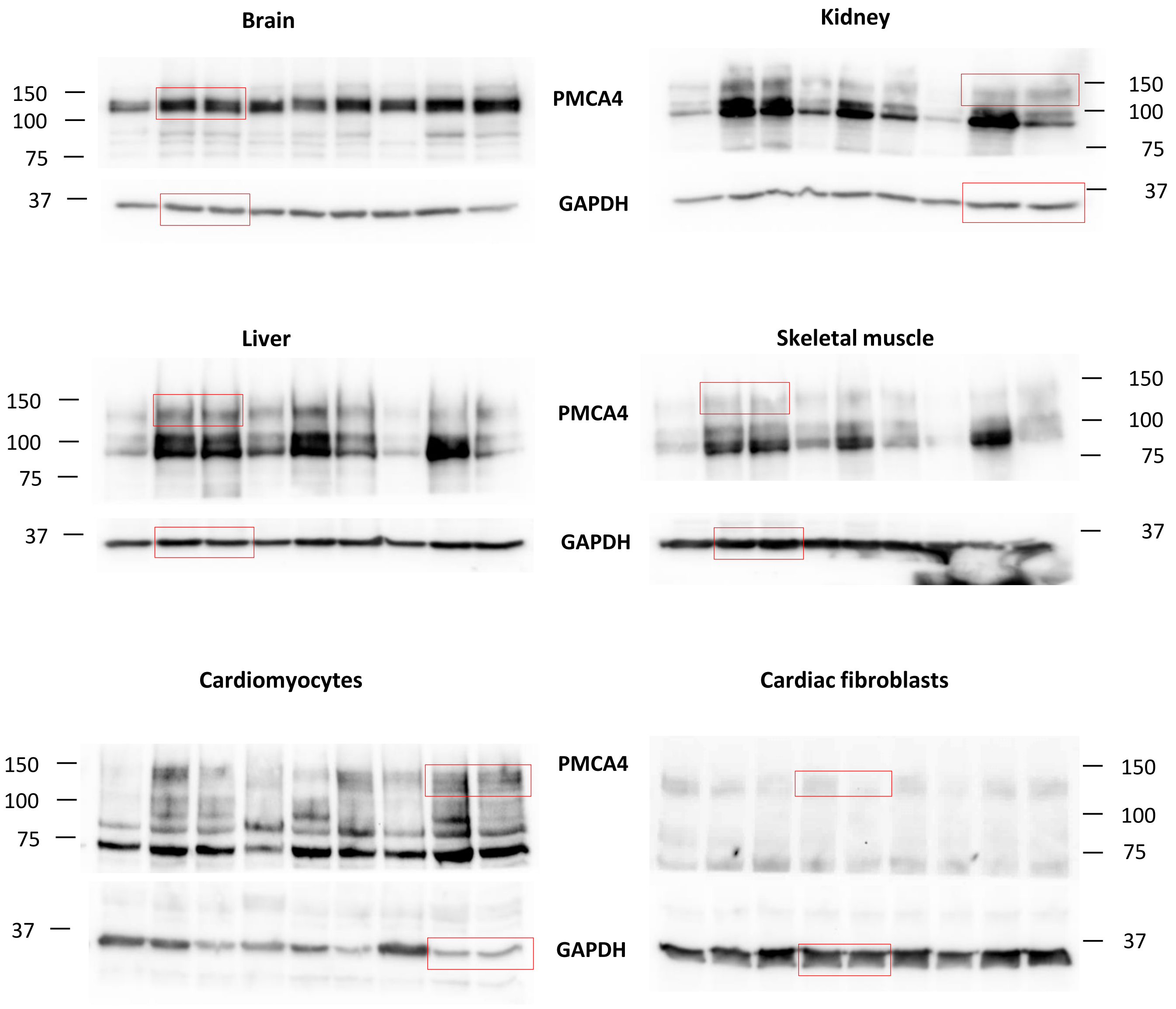

Figure 5J

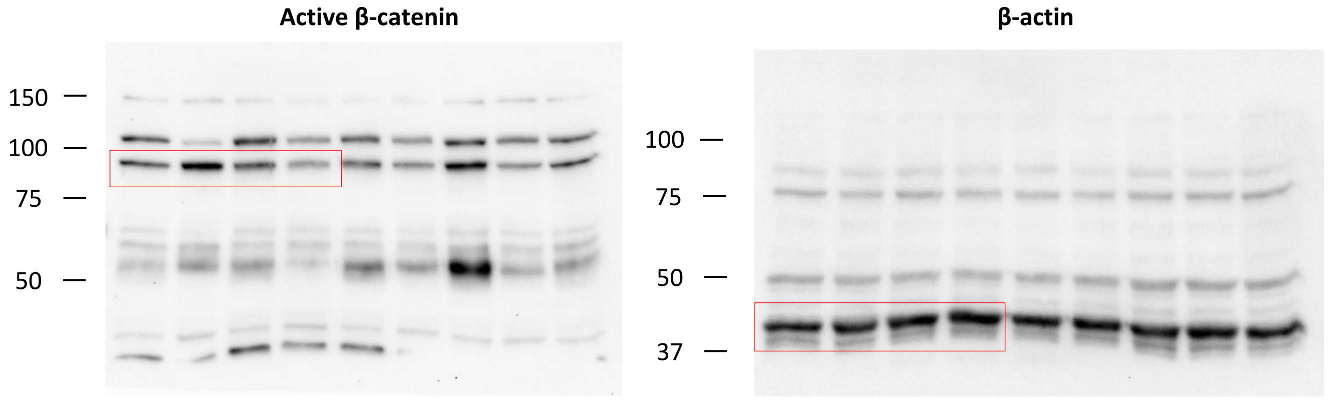

Figure 7G

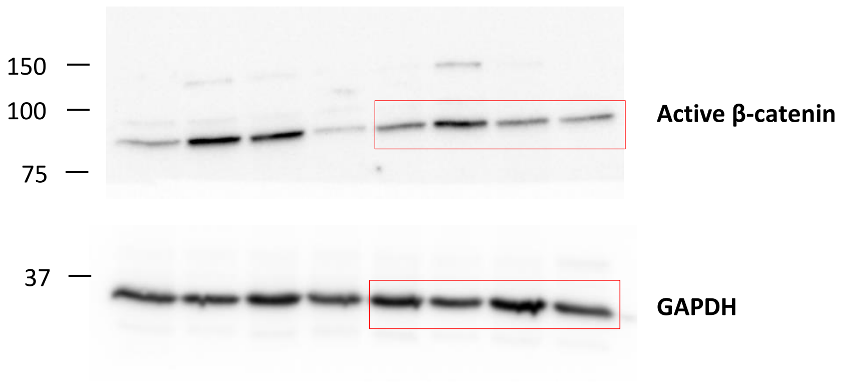

Supplementary figure 5A

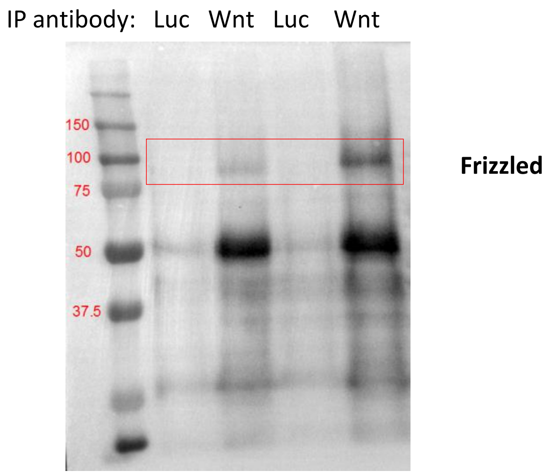

Supplementary figure 5F

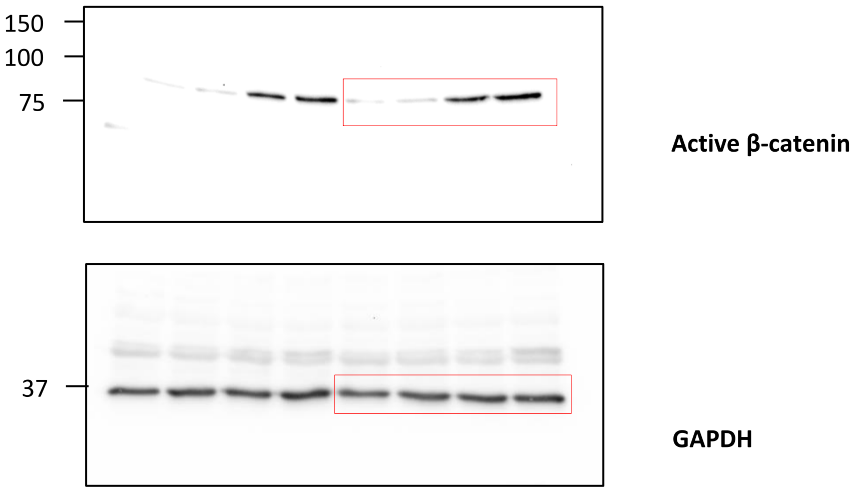

Supplementary figure 13A

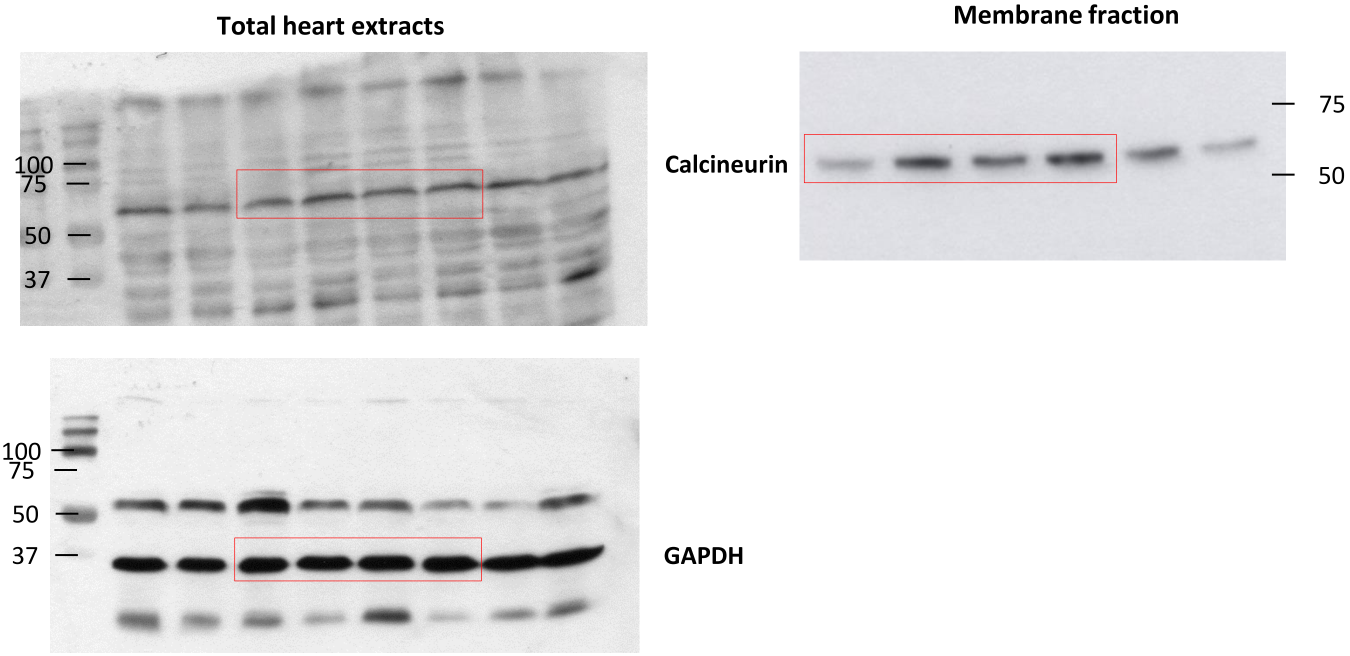

Supplementary figure 14 Full western blots from which the cropped images in figures 1, 3, 4, 5 and 7 in the main paper and supplementary figures 5 and 13 were taken. The red boxes indicate the cropped area. *PMCA4*^{cko} and *PMCA4*^{fko} samples were run on the same gel for cardiomyocyte and cardiac fibroblast blots shown in figures 4B and 5B

Supplementary Table 1 Echocardiographic parameters obtained from wild type and *PMCA4*$^{-/-}$ mice following TAC or sham surgery

	WT sham (n=10)	WT TAC (n=10)	*PMCA4*$^{-/-}$ sham (n=10)	*PMCA4*$^{-/-}$ TAC (n=10)
LVEDD (mm)	4.16 ± 0.10	4.27 ± 0.17	4.0 ± 0.09	3.80 ± 0.08*
LVESD (mm)	3.03 ± 0.14	3.01 ± 0.17	2.99 ± 0.09	2.71 ± 0.08
Wall thickness (mm)	0.80 ± 0.03	1.04 ± 0.05$^{\#}$	0.84 ± 0.03	0.89 ± 0.03*
LV mass (mg)	118.5 ± 3.5	187.5 ± 23.5$^{\#}$	120.1 ± 8.7	127.8 ± 9.6*

LVEDD, Left ventricular end diastolic dimension; LVESD, Left ventricular end systolic dimension; Wall thickness, average of interventricular septal and posterior wall thickness in diastole; LV mass, calculated left ventricular mass

*P < 0.05 vs WT TAC; $^{\#}$P<0.05 vs WT sham

Supplementary Table 2 Echocardiographic parameters obtained from *PMCA4*^{cko} and control mice following TAC or sham surgery

	Control sham (n=7)	Control TAC (n=9)	*PMCA4*^{cko} sham (n=6)	*PMCA4*^{cko} TAC (n=7)
LVEDD (mm)	4.13 ± 0.09	3.88 ± 0.15	3.70 ± 0.15	4.14 ± 0.16
LVESD (mm)	3.05 ± 0.12	2.80 ± 0.12	2.62 ± 0.09	3.18 ± 0.12
Wall thickness (mm)	0.81 ± 0.02	1.15 ± 0.07$^{\#}$	0.83 ± 0.05	1.03 ± 0.02§
LV mass (mg)	121.0 ± 5.7	187.6 ± 15.7$^{\#}$	103.4 ± 6.9	173.1 ± 13.8§

LVEDD, Left ventricular end diastolic dimension; LVESD, Left ventricular end systolic dimension; Wall thickness, average of interventricular septal and posterior wall thickness in diastole; LV mass, calculated left ventricular mass; *PMCA4*$^{flox/flox}$ littermates were used as controls

$^{\#}$P<0.05 vs control-sham; §P<0.05 vs *PMCA4*^{cko} sham

Supplementary Table 3 Echocardiographic parameters obtained from *PMCA*^{fko} and control mice following TAC or sham surgery

	Control sham (n=4)	Control TAC (n=5)	*PMCA4*^{fko} sham (n=4)	*PMCA4*^{fko} TAC (n=4)
LVEDD (mm)	4.39 ± 0.27	4.19 ± 0.29	4.38 ± 0.14	3.76 ± 0.35
LVESD (mm)	3.22 ± 0.19	3.06 ± 0.27	3.14 ± 0.08	2.70 ± 0.42
Wall thickness (mm)	0.87 ± 0.03	1.42 ± 0.06$^{\#}$	0.91 ± 0.03	1.26 ± 0.01§,*
LV mass (mg)	144.9 ± 8.1	258.4 ± 13.8$^{\#}$	153.6 ± 6.3	196.2 ± 24.7§,*

LVEDD, Left ventricular end diastolic dimension; LVESD, Left ventricular end systolic dimension; Wall thickness, average of interventricular septal and posterior wall thickness in diastole; LV mass, calculated left ventricular mass; *PMCA4*$^{flox/flox}$ littermates were used as controls

$^{\#}$P<0.05 vs control-sham; §P<0.05 vs *PMCA4*^{fko} sham; *P<0.05 vs control TAC

Supplementary Table 4 Phenotypic analysis of *PMCA4*$^{-/-}$ mice

Parameter	Age (weeks)	WT	*PMCA4*$^{-/-}$	P value
General parameters				
Body weight (g)	17	31.9±0.72	31.5±0.67	NS
Body temperature (^{0}C)	17	36.48±0.24	36.65±0.26	NS
Food intake (g per mouse per day)	17	4.3±0.3	4.7±0.5	NS
Blood chemistry				
Urea (mmol per l)	19	8.9±0.71	7.2±0.37	NS
Creatinine (µmol per l)	19	34±1.6	31±1.4	NS
Uric acid (µmol per l)	19	95±16.2	87±12.6	NS
Sodium (mmol per l)	19	149±0.7	146±2.1	NS
Potassium (mmol per l)	19	4.9±0.31	5.4±0.44	NS
Chloride (mmol per l)	19	111±0.7	109±2.3	NS
Bicarbonate (mmol per l)	19	19±0.7	19±0.6	NS
Total proteins (g per l)	19	63±0.6	63±0.8	NS
Albumin (g per l)	19	29±0.5	30±0.6	NS
Calcium (mmol per l)	19	2.08±0.03	2.03±0.04	NS
Phosphorus (mmol per l)	19	2.16±0.25	2.34±0.17	NS
Magnesium (mmol per l)	19	1.18±0.04	1.12±0.05	NS
Iron (µmol per l)	19	23.1±0.8	24.9±0.56	NS
AST (U per l)	21	94±26	89±14	NS
ALT (U per l)	21	38±15.6	29±5.9	NS
ALP (U per l)	21	100±4.9	97±6.6	NS
α-amylase (U per l)	21	714±30.4	765±30.5	NS
Total cholesterol (mmol per l)	19	3.01±0.09	3.22±0.05	NS
HDL-cholesterol (mmol per l)	19	2.17±0.05	2.33±0.12	NS
LDL-cholesterol (mmol per l)	19	0.39±0.02	0.38±0.02	NS
Triglycerides (mmol per l)	19	0.93±0.12	0.97±0.14	NS
Free fatty acids (mEq per l)	19	1.07±0.16	1.06±0.04	NS
T3 thyroid hormone (nmol per l)	26	0.39±0.07	0.42±0.14	NS
T4 thyroid hormone (nmol per l)	26	33.1±2.6	34.6±3.4	NS
Blood parameters				
White blood cell (X10^{3} per µl)	19	5.4±0.72	6.2±0.77	NS
Red blood cell (X10^{3} per µl)	19	8.74±0.64	9.34±0.33	NS
Hemoglobin (g per dl)	19	13.8±1.06	15.1±0.52	NS
Hematocrit (%)	19	41.7±3.36	45.0±1.83	NS
Platelets (X10^{3} per µl)	19	1342±104	1543±112	NS
Prothrombin time (sec)	30	10.2±0.3	10.4±0.1	NS
Fibrinogen (g per l)	30	2.2±0.1	2.09±0.1	NS
Immunology				
IgG (µg per ml)	21	1231±170	1242±279	NS
IgM (µg per ml)	21	777±138	835±113	NS

IgA (µg per ml)	21	148±15	382±99	NS
Indirect calorimetry				
O_2 consumption – VO_2 mean 0h-6h (ml per kg per Hr)	20	1321±30	1278±45	NS
Respiratory exchange ratio – mean 0h-6h	20	0.95±0.02	0.95±0.02	NS
Heat production - mean 0h-6h (kcal per Hr)	20	0.52±0.02	0.49±0.03	NS
Dexascan				
Fat tissue (g)	22	7.0±0.5	6.2±0.4	NS
Lean tissue (g)	22	25.2±0.4	24.3±0.7	NS
Bone mineral density (g per cm^2)	22	0.051 ±0.0006	0.051 ±0.0004	NS
Bone mineral content (g)	22	0.40±0.01	0.42±0.01	NS
Bone area (cm^2)	22	7.9±0.2	8.1±0.1	NS
X-ray				
Width 3rd lumbar vertebra (mm)	22	3.0±0.03	3.0±0.03	NS
Height 3rd lumbar vertebra (mm)	22	3.23±0.08	3.31±0.08	NS
Length ulna (mm)	22	15.06±0.14	14.93±0.12	NS
Length left metacarpal (mm)	22	3.2±0.09	3.25±0.08	NS
Width femur (mm)	22	1.6±0.04	1.57±0.05	NS
Length femur (mm)	22	15.42±0.22	15.25±0.2	NS
Nervous system				
Rotarod (sec)	17	110.6±10.6	97.6±16.3	NS
String test (sec)	17	2.7±0.6	2.8±0.3	NS
Grip strength	17	7.5±0.2	8.2±0.3	NS
Pain sensitivity<br>• Tail flick (sec)<br>• Hot plate – jump (sec)	17	5.8±0.8<br>180.0±0.0	6.0±0.6<br>166.7±9.7	NS<br>NS
Shock threshold – flinch (mA)	17	0.09±0.01	0.08±0.01	NS
Open field test<br>• Rears<br>• Entries into centre	17	117±26.5<br>61±17	80±17.4<br>25±10	NS<br>NS
Tail suspension (sec)<br>• Immobility<br>• Latency to despair	17	190±11.3<br>64.1±5.6	213±13<br>61.5±9.6	NS<br>NS

AST, aspartate transaminase; ALT, alanine transaminase; ALP, alkaline phosphatase; ABR, auditory brainstem response; OMR optomotor response; %BW, percentage of total body weight; NS, not significant.
